# Collagen IV of basement membranes: IV. Adaptive mechanism of collagen IV scaffold assembly in *Drosophila*

**DOI:** 10.1016/j.jbc.2023.105394

**Published:** 2023-10-27

**Authors:** Jacob A. Summers, Madison Yarbrough, Min Liu, W. Hayes McDonald, Billy G. Hudson, José C. Pastor-Pareja, Sergei P. Boudko

**Affiliations:** 1 Aspirnaut Program, Vanderbilt University Medical Center, Nashville, Tennessee, USA; 2 School of Life Sciences, Tsinghua University, Beijing, China; 3 Proteomics Laboratory, Mass Spectrometry Research Center, Vanderbilt University, Nashville, Tennessee, USA; 4 Department of Biochemistry, Vanderbilt University, Nashville, Tennessee, USA; 5 Division of Nephrology and Hypertension, Department of Medicine, Vanderbilt University Medical Center, Nashville, Tennessee, USA; 6 Center for Matrix Biology, Vanderbilt University Medical Center, Nashville, Tennessee, USA; 7 Department of Pathology, Microbiology, and Immunology, Vanderbilt University Medical Center, Nashville, Tennessee, USA; 8 Department of Cell and Developmental Biology, Vanderbilt University, Nashville, Tennessee, USA; 9 Vanderbilt-Ingram Cancer Center, Nashville, Tennessee, USA; 10 Vanderbilt Institute of Chemical Biology, Vanderbilt University, Nashville, Tennessee, USA; 11 Tsinghua-Peking Center for Life Sciences, Beijing, China; 12 Institute of Neurosciences, Consejo Superior de Investigaciones Científicas-Universidad Miguel Hernández, San Juan de Alicante, Spain

**Keywords:** collagen IV, basement membrane, NC1 domain, *Drosophila*, chloride, divalent cations, protein self-assembly, crystal structure, extracellular matrix, protein evolution

## Abstract

Collagen IV is an essential structural protein in all metazoans. It provides a scaffold for the assembly of basement membranes, a specialized form of extracellular matrix, which anchors and signals cells and provides microscale tensile strength. Defective scaffolds cause basement membrane destabilization and tissue dysfunction. Scaffolds are composed of α-chains that coassemble into triple-helical protomers of distinct chain compositions, which in turn oligomerize into supramolecular scaffolds. Chloride ions mediate the oligomerization via NC1 trimeric domains, forming an NC1 hexamer at the protomer–protomer interface. The chloride concentration–“chloride pressure”–on the outside of cells is a primordial innovation that drives the assembly and dynamic stabilization of collagen IV scaffolds. However, a Cl-independent mechanism is operative in Ctenophora, Ecdysozoa, and Rotifera, which suggests evolutionary adaptations to environmental or tissue conditions. An understanding of these exceptions, such as the example of *Drosophila*, could shed light on the fundamentals of how NC1 trimers direct the oligomerization of protomers into scaffolds. Here, we investigated the NC1 assembly of *Drosophila*. We solved the crystal structure of the NC1 hexamer, determined the chain composition of protomers, and found that *Drosophila* adapted an evolutionarily unique mechanism of scaffold assembly that requires divalent cations. By studying the *Drosophila* case we highlighted the mechanistic role of chloride pressure for maintaining functionality of the NC1 domain in humans. Moreover, we discovered that the NC1 trimers encode information for homing protomers to distant tissue locations, providing clues for the development of protein replacement therapy for collagen IV genetic diseases.

The basement membrane (BM) is a specialized form of extracellular matrix mainly composed of collagen, laminin, perlecan, and nidogen (1, 2, 3, 4, 5, 6, 7). Evolved, the appearance of the BM advanced the multicellularity of animals to a level of highly diverse and specialized tissues and organs (8, 9, 10, 11). Collagen IV is a core protein in organizing the BM structure, maintaining integrity, and performing numerous functions (12, 13). The protomer of collagen IV combines three α chains into a triple-helical structure. The C-terminal noncollagenous domain (NC1) is responsible for chain selection and trimerization inside the cells (Fig. 1) (14, 15, 16, 17). Once outside the cells, protomers assemble into a scaffold *via* interactions including those of the N-terminal 7S regions and the C-terminal NC1 domains (Fig. 1) (18, 19, 20, 21). The NC1 hexamer is then further stabilized by covalent sulfilimine bonds connecting NC1 domains from two opposite protomers (Fig. 1) (22, 23, 24, 25). In mammals, the NC1 hexamer assembly is triggered by a high concentration of extracellular chloride ions outside the cell (26, 27). Namely, 12 Cl^-^ ions are involved in the assembly and stabilization of the NC1 hexamer, forming a ring at the interface between two protomers (28, 29). The chloride-dependent mechanism evolved early in cnidarians and has been preserved up to mammals (28, 30). Indeed, we found experimentally that the Cl-dependent mechanism extended from basal cnidarians to mammals except for Ctenophora, Ecdysozoa, and Rotifera (30). We concluded that the chloride concentration“chloride pressure”–on the outside of cells is a primordial innovation that drives the assembly and dynamically stabilizes and maintains collagen IV scaffolds. The exceptions suggest evolutionary adaptations of assembly and stability to environmental or tissue conditions. An understanding of such an exception, as in the example of *Drosophila*, could shed light on the fundamentals of how NC1 trimers direct the oligomerization of protomers into scaffolds on the outside of cells.

**Figure 1:**
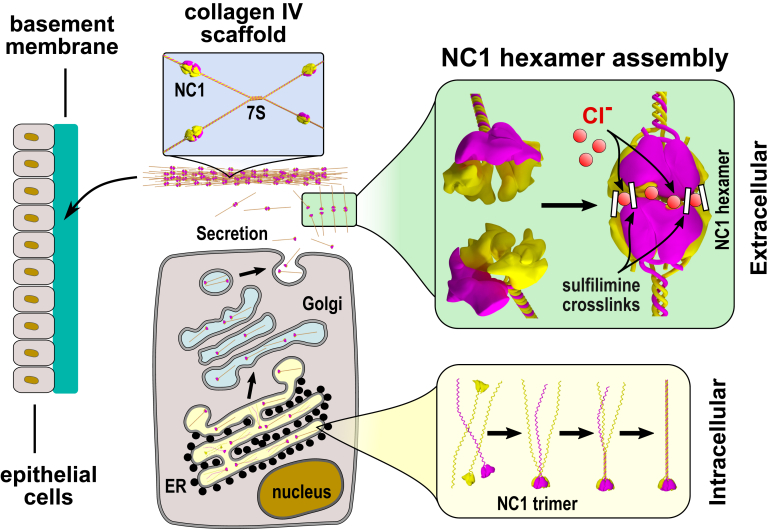
**Assembly steps of collagen IV protomers and scaffold in mammals.** Translated chains of collagen IV are being assembled into trimeric protomers inside the endoplasmic reticulum (ER). Single chains are brought together and aligned by the C-terminal noncollagenous domain 1 (NC1), which initiates the folding of the collagenous part in a zipper-like manner. The scaffold assembly begins when collagen IV molecules are secreted outside the cell. The key step is the hexamer assembly between two NC1 trimers, which is triggered by a high concentration of chloride ions outside the cell in mammals. After the hexamer is formed and stabilized by 12 chloride ions the structure gets further reinforced by sulfilimine bonds connecting opposite NC1 subunits from two trimers. The 7S dodecamer assembly of four protomers together with the NC1 hexamer formation are two major steps for building a continuous collagen IV scaffold. The role of chloride ions in the assembly of the NC1 hexamer in nonmammalian species is yet unknown.

*Drosophila* has been used productively as a model organism for over a century to study a range of diverse processes in biology and medicine, including genetics and inheritance, embryonic development, organ regeneration, learning, behavior, and aging due to its simplicity and availability of multiple research tools (31, 32, 33). Remarkably, the insect nephrocyte has anatomical, molecular, and functional similarities to the glomerular podocyte, a cell in the vertebrate kidney that forms the main size-selective barrier as blood is ultrafiltered to make urine (34). Diabetes can be induced in *Drosophila* following mechanisms similar to those in humans and its renal system has been used as a model for identifying drug targets for diabetic nephropathy and screening the potential drugs for their efficacy (35). *Drosophila* has a simpler toolbox of proteins used to build the BM (36), making it a nonredundant system and simplifying functional analysis. Unlike humans which have 28 types of collagens encoded by 47 genes, *Drosophila* contains only two collagens encoded by three genes. One of these genes encodes multiplexin (scarce BM-associated collagen), whereas the other two are collagen IV genes, *Cg25c* (37, 38) and *Viking* (39) (Fig. 2) sometimes referred to as coding α1 and α2 chains based on the chronology of discovery. In contrast, humans contain six collagen IV chains, *COL4A1* through *COL4A6*, which were shown to form three types of scaffolds, that is, α121, α345, and α121/α565 (16, 17, 20, 40, 41, 42). It has been shown previously that the NC1 domain in *Drosophila* forms a hexamer, whose quaternary structure is further reinforced by sulfilimine covalent bonds (23), unique to collagen IV, and discovered in numerous animal species (25). Nevertheless, our knowledge about the chain composition and scaffolding properties of *Drosophila* collagen IV remains controversial with several reports, suggesting the existence of homotrimeric molecules (43, 44).

**Figure 2:**
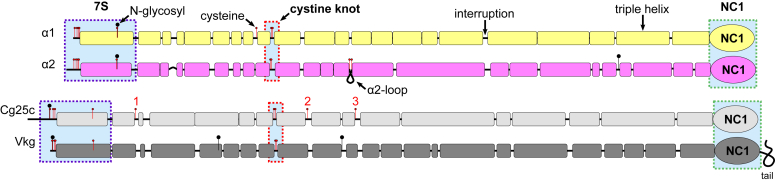
**Comparison of human and *Drosophila* collagen IV chains.** Schematic presentation of α1 and α2 chains of human and Cg25c and Vkg chains of *Drosophila*. They share a common organization, which includes the 7S domain, the cystine knot, the NC1 domain, and multiple triple-helical segments alternated with interruptions. Nevertheless, overall lengths of the collagenous domains are longer in *Drosophila*, the Vkg chain has an extension of the NC1 domain ("tail”), and there is a significant variability in the sizes and locations of the triple-helical segments. *Bars* represent triple-helical segments. *Black lines* represent nontriple-helical sequences/interruptions. Cysteines are shown as *red pins*, and potential sites for N-glycosylation of asparagine are shown as *black pins*. Conservative regions are shaded with *light blue boxes* and labeled 7S, cystine knot, and NC1. A close-in view of the 7S region is shown in Fig.S1. A cystine knot is characterized by a pair of cysteines within an interruption. Vkg chain has an appendix following the NC1 domain (labeled as tail). Note, that the Cg25c chain contains three single cysteines, labeled 1, 2, and 3, not found in human chains. The longest interruption flanked by two cysteines in the human α2 chain (labeled as α2-loop) is not found in any *drosophila* chains. Also note different distributions of potential N-glycosylation sites. NC1, noncollagenous domain 1.

Here, we solved the crystal structure of NC1 hexamer, determined the chain composition, and found that *Drosophila* adapted an evolutionarily unique mechanism for scaffold assembly requiring divalent cations. Furthermore, we found that the NC1 trimer encodes information for homing to distant tissue locations in scaffold assembly, providing clues for developing protein replacement therapy in humans.

## Results

### *Drosophila* sequences share common and have their unique traits of mammalian collagen IV

Primary sequences of Cg25c and Vkg chains contain specific sequences, which are hallmarks of collagen IV, that is, a 7S-like region, a cystine knot (45), and the NC1 domain (Fig. 2). The 7S-like region though has three N-terminal cysteines instead of four in human and the glycosylation sites are swapped (Fig. S1). Given the antiparallel arrangement of chains within the 7S structure, the swapped glycosylation sites could still have similar structural and functional impacts (19, 46, 47). Distribution of predicted triple helical segments and interruptions along the collagenous domains of human and *Drosophila* chains do not appear to have common patterns, which would otherwise suggest the assignment of Cg25c and Vkg to α1- or α2-like chains. Moreover, the collagenous domains in *Drosophila* are longer (Fig. 2). An α2-specific loop (48) found in all vertebrate chains and characterized by the longest interruption flanked by two cysteines is missing in either *Drosophila* chain. Vkg also has a unique tail following the NC1 domain, which is not found in Cg25c or any vertebrate chain. The function of this tail remains unknown. In humans and mice, a mutation causing extension of the NC1 domain that somewhat mimics the “tail” leads to pathologies (49). Vkg tails of different species of the *Drosophila* genus show some conservation only within the last ∼25 residues (Fig. S2), suggesting its functional role is yet to be discovered.

Interestingly, in humans, there is a single unpaired cysteine within the collagenous domain of the α1 chain and none in α2 (Fig. 2). Given the α1-α1-α2 composition of the protomer, this single cysteine can form a disulfide bond with the same cysteine of the second α1 chain, thus avoiding reactive sulfhydryl groups on the surface of the assembled collagenous domain. In *Drosophila*, only Cg25c has three unpaired cysteines along the collagenous domain (Fig. 2), which suggests the Cg25c-Cg25c-Vkg composition to pair those cysteines into three interchain disulfide bridges and avoid reactive sulfhydryl groups on the surface of the collagenous domain. Thus, primary sequence analysis suggests the Cg25c-Cg25c-Vkg composition of collagen IV in *Drosophila*. In accord, previous genetic data also suggested that two Cg25c chains interact in forming the trimer (50).

### Chain clustering into NC1 α1- and α2-like groups

Collagen IV NC1 chains were found to cluster into α1- and α2-like groups, where vertebrate chains α1, α3, and α5 form an α1-like group and α2, α4, and α6 comprise an α2-like group (51). All known mammalian protomer compositions follow the rule of combining two α1-like chains and one α2-like, that is, α112, α345, and α556 (16, 17, 20, 40, 41, 42). While the *Drosophila* Cg25c chain clustered into the α2-like group, Vkg stood out of both groups (Fig. S3). When *Drosophila* chains were analyzed for sequence identity with human chains, Cg25c had higher scores toward α2-like chains, whereas Vkg had higher identity toward α1-like chains (Fig. S4). Although Cg25c has a higher identity with α2-like chains than Vkg it also has a higher identity to α1-like chains than Vkg, thus placing Vkg again in an evolutionarily most distinct chain. Given somewhat confusing predictions for chain identities in *Drosophila*, we had to explore all possible combinations of chains including homo and hetero variants to find out the fruit fly’s own rule for trimer composition (*vide infra*).

### Isolation of endogenous NC1 hexamer containing both Cg25c and Vkg chains

We used *Drosophila* pellet to extract and purify endogenous NC1 hexamer as described in Experimental procedures. After collagenase treatment soluble material was initially fractionated by size-exclusion chromatography (SEC) (Fig. S5). Analysis of fractions on the SDS-PAGE suggested that the NC1 hexamer was contained within the 12.99 ml peak (Fig. S5), similar to mammalian NC1 hexamers. Of note, the NC1 hexamer revealed SDS resistance when SDS samples were prepared at room temperature without boiling (30). Upon boiling, the hexamer dissociates into dimers and monomers (Fig. S5). We did not detect any “tailed” version of the Vkg chain in fractions with apparent higher macromolecular sizes, which would also contain Cg25c NC1 bands of normal size (Fig. S5). The NC1 fraction was pooled and further purified by anion-exchange chromatography and additionally run through a hydroxyapatite column to remove residual impurities (Fig. S6). The in-gel trypsin or chymotrypsin digestion and tandem mass spectrometry (MS/MS) analysis detected peptide fragments of the NC1 domain from both Cg25c and Vkg chains (Fig. S7), but not the “tail” sequence of Vkg (Fig. S2). Collectively, *Drosophila* collagen IV does form the hexamer, which contains both Cg25c and Vkg chains; and the Vkg “tail” sequence (not found in mammals) is not an integral part of the NC1 hexamer.

### NC1 monomers assemble into transient heterotrimers *in vitro*

**Figure 3:**
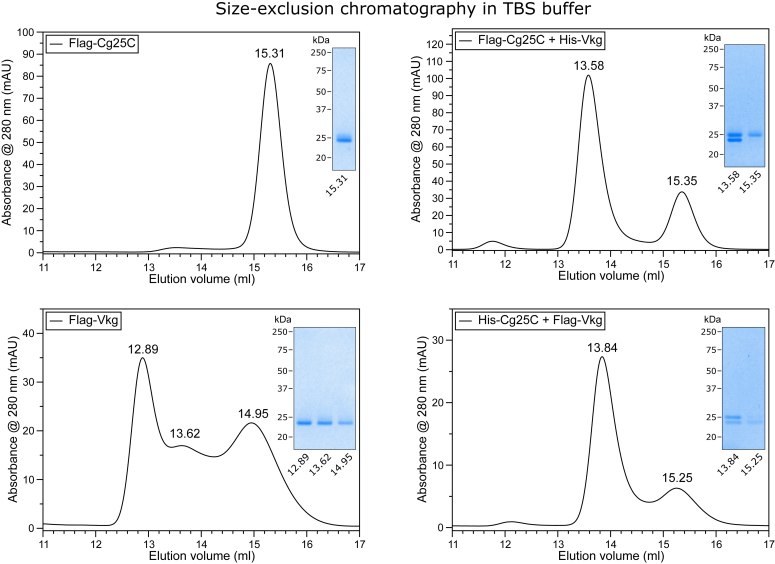
**Size-exclusion chromatography of affinity-purified NC1 domains in the presence of chloride.** Chromatograms of individually expressed Flag-Cg25c and Flag-Vkg NC1 domains and coexpressed combinations of Flag-Cg25c with His-Vkg NC1 and His-Cg25c and Flag-Vkg NC1. Major peaks were then analyzed on SDS-PAGE. Cg25c eluted as a single monomer peak, whereas Vkg had a broad distribution of different oligomers ranging apparently from hexamer (12.89 ml, compared to 12.95 ml of tissue-extracted NC1 hexamer) to trimer (13.62 ml) and to dimer-monomer (∼15 ml). When coexpressed, Cg25c and Vkg chains revealed the appearance of peaks at 13.58 and 13.84 ml corresponding to a trimer formation. Double bands are observed for trimer fractions on the gel stained with Coomassie corresponding to Cg25c (*upper*) and Vkg (*lower*) NC1 domains. Overall, the coexpression of Cg25c and Vkg resulted in the formation of a heterotrimer but not a hexamer. NC1, noncollagenous domain 1.

To analyze the self-assembly properties of the NC1 chains, we performed a series of recombinant expressions of Cg25c and Vkg monomers (Fig. S8). The Vkg construct was made without the “tail” sequence as it was not found to be an integral part of the hexamer (*vide supra*). Each monomer contained a signal peptide for secretion into the medium and either a FLAG- or His-tag for purification purposes. The secreted proteins were purified from media using anti-FLAG resin and analyzed by SEC. Individually expressed Cg25c NC1 was found to be predominantly a monomer, whereas Vkg NC1 revealed a mixture of oligomers ranging from a monomer to an apparent hexamer (Fig. 3) resembling the native hexamer peak (Fig. S6). Coexpression of Cg25c and Viking NC1 domains revealed the formation of an apparent trimer (Fig. 3) with both chains detected as double bands by SDS-PAGE (Fig. 3 inserts). To our knowledge formation of a stable NC1 trimer lacking triple helical sequences was never observed before. Of note, the purification and analysis were performed at a high Cl^-^ concentration at which mammalian NC1 monomers can form a hexamer but were never detected as a trimer (26, 52). We also found that the depletion of Cl^-^ does not affect the oligomer state (Fig. S9) though mammalian NC1 hexamers would dissociate into monomers under the same experimental conditions (26).

The ratio of Cg25c and Viking chains in purified trimers seemed to range from ∼2:1 to ∼1:2 as estimated from gel band intensities (Fig. 3 inserts). It suggested the existence of both Cg25c_2_Vkg_1_ and Vkg_2_Cg25c_1_ heterotrimers. Nevertheless, we found that these trimers are transient, that is, they have a low-affinity association/dissociation equilibrium. Once captured by a chain-specific tag, a leakage of another chain bearing a different tag was detected (Fig. S10). Also, SEC reanalysis of the heterotrimer purified by two serial rounds of chain-specific affinity chromatographies still revealed the presence of monomers (Fig. S10). Collectively, the transient nature of the NC1 trimer suggests that a triple helical region adjacent to the NC1 domain contributes if not to selection but at least to the stabilization of a specific composition of chains. At this point, we still could not exclude the existence of homotrimer compositions of collagen IV in *Drosophila*.

### Transgenic expression, secretion, and deposition of NC1 single-chain trimers

**Figure 4:**
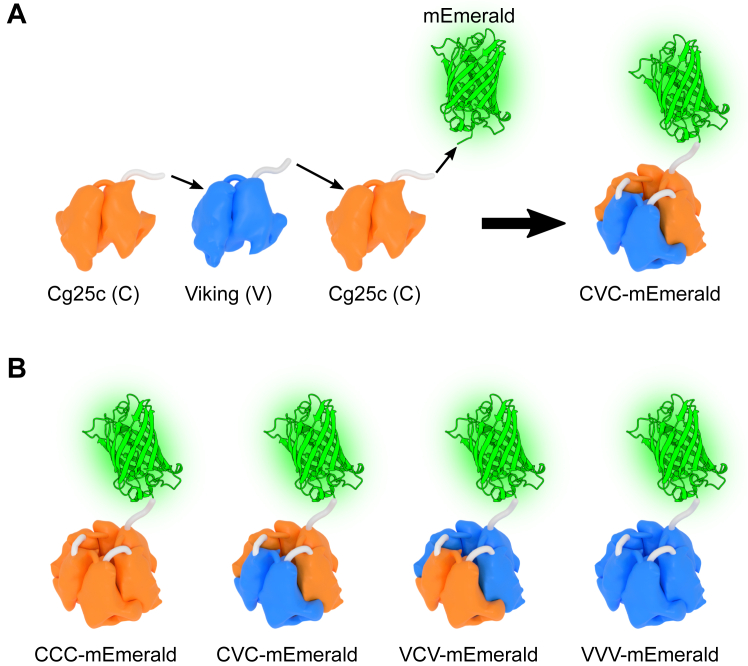
**Single-chain constructs of *Drosophila* NC1 trimer with fluorescent protein fusion.** Cg25c (C), Vkg (V) NC1, and fluorescent protein mEmerald sequences were assembled into a single DNA construct for transgenic experiments and recombinant expression. *A*, the CVC construct combines Cg25c, Vkg, Cg25c, and mEmerald sequences linked with artificial linkers. *B*, variants CCC-mEmerald, CVC-mEmerald, VCV-mEmerald, and VVV-mEmerald were used as transgenes and for recombinant expression. The single-chain trimers are designed in a way to control composition and mimic a stabilizing role of the triple helix of the native protomers. The absence of the collagenous domain allows to focus on the functionality of the NC1 domain, while the fusion of the fluorescent probe facilitates the detection. NC1, noncollagenous domain 1.

**Figure 5:**
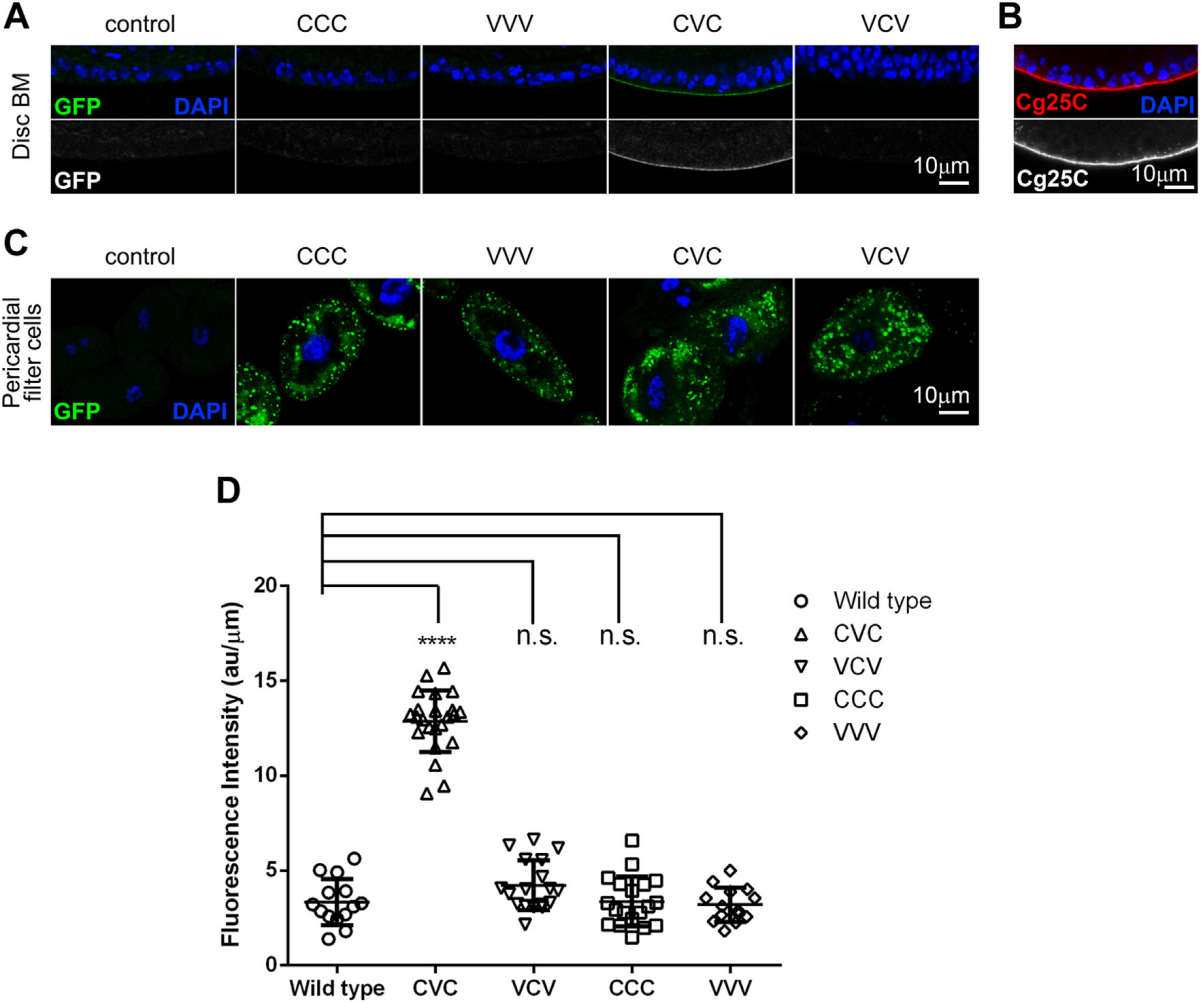
**Expression, secretion, and BM deposition of GFP-tagged NC1 trimeric variants.** All four constructs were successfully expressed and secreted to the hemolymph but only CVC was deposited to the BM. *A*, confocal images of wing imaginal discs from control larvae and larvae expressing mEmerald GFP-tagged (*green*) NC1 trimeric constructs in the fat body. Nuclei stained with DAPI (*blue*). The GFP channel is shown separately in *white* on *lower panels*. The presence of a GFP signal indicates the incorporation of CVC into the BM. *B*, confocal image of a WT wing imaginal disc stained with anti-Cg25c (*red*) to reveal the BM. Nuclei stained with DAPI (*blue*). The anti-Cg25c channel is shown separately in *white* on the *lower panel*. *C*, confocal images of pericardial filter cells from control larvae and larvae expressing mEmerald GFP-tagged (*green*) NC1 trimeric constructs in the fat body. Nuclei stained with DAPI (*blue*). The GFP channel is shown separately in *white* on the *lower panels*. The presence of a GFP signal in filter cells indicates efficient secretion into the hemolymph. *D*, quantification of BM incorporation of different NC1 trimeric constructs measured from images like those in (*A*). The significance of differences with the control in unpaired *t* tests is represented. *p* > 0.05: n.s; *p* < 0.0001: ∗∗∗∗. The scale bar represents 10 μm. BM, basement membrane; DPI, 4′,6-diamidino-2-phenylindole; NC1, noncollagenous domain 1.

To examine which composition of NC1 trimer is biologically active, we expressed all possible combinations of NC1 as single-chain trimer transgenes in *Drosophila melanogaster*. A modified green fluorescent protein, mEmerald, was tagged to the C terminus of each variant for detection (Fig. 4). The trimers were designed using a single-chain approach developed for human NC1 domains earlier (28, 53). Instead of using short and rigid 3-residue linkers between NC1 monomers (28), we used elongated and flexible 7-residue linkers to avoid any possible steric hindrances due to possible structural variations between human and *Drosophila* structures. With two genes encoding collagen IV chains in *Drosophila*, that is*, Cg25c* (C) and *Viking* (V) (37, 38, 39), all four possible variants: CCC-mEmerald, CVC-mEmerald, VCV-mEmerald, and VVV-mEmerald were designed (Figs. 4 and S11–S14) and initially probed for folding and secretion in a mammalian expression system. All four were secreted, affinity purified using anti-FLAG resin, and analyzed by SEC. CCC-, CVC-, and VCV-mEmerald were found to be predominantly “monomeric” (single-chain trimer), while VVV-mEmerald demonstrated a tendency to form higher oligomers (Fig. S15). When expressed in *Drosophila* larvae using the GAL4-UAS binary expression system, all transgenes were efficiently secreted into the hemolymph, as revealed by the presence of Emerald GFP signal in the kidney-like, hemolymph-filtering pericardial cells (Fig. 5). However, only CVC-mEmerald was found to be incorporated into the BM (Fig. 5). Thereto, an NC1 trimer is sufficient for targeting and incorporation into the BM. Whether it is solely achieved *via* NC1 hexamer assembly with a full-length protomer localized in the BM or whether there are specific ligands for hosting the NC1 trimer remains to be elucidated. Collectively, the Cg25c:Cg25c:Vkg combination of the NC1 trimer was found to be biologically functional.

### NC1 trimers spontaneously form hexamers *in vitro*

**Figure 6:**
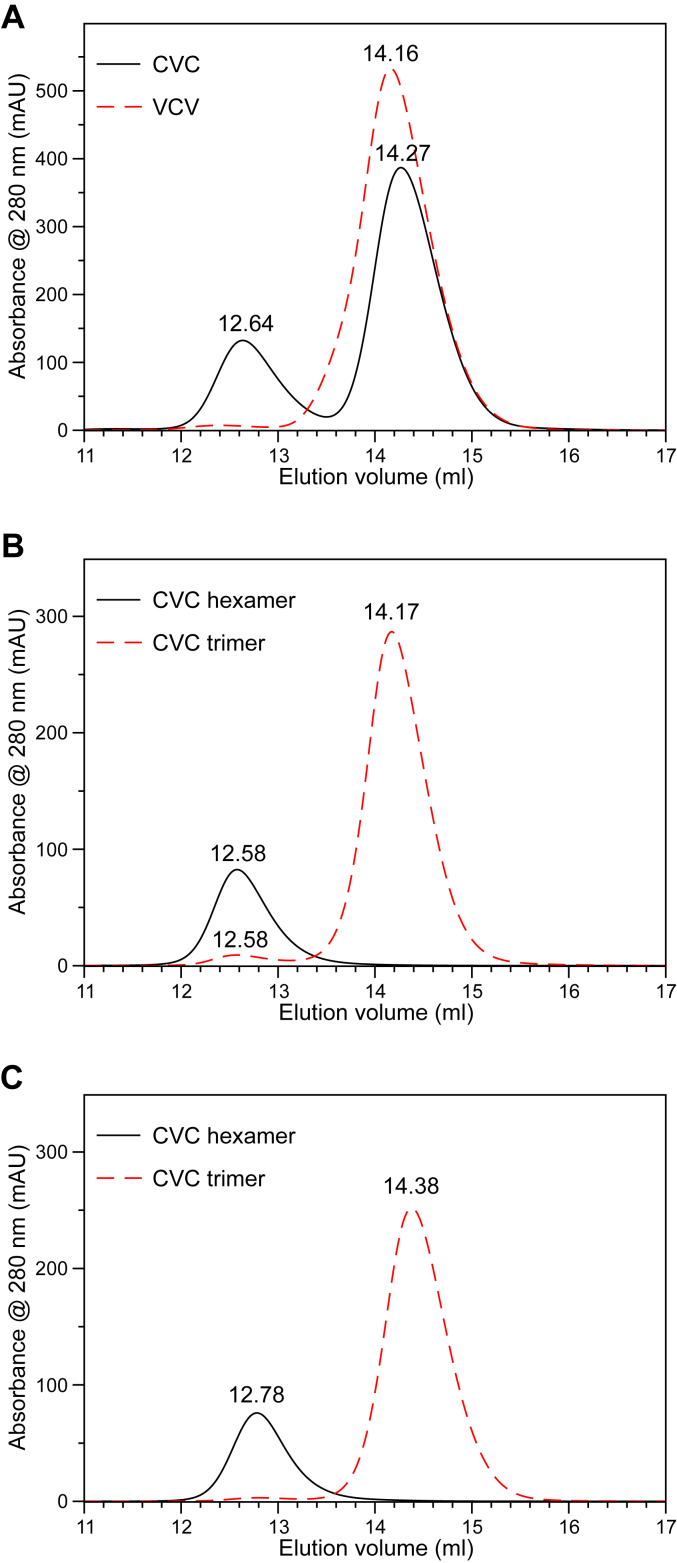
**Single-chain CVC trimer “spontaneously” assembles into the hexamer.** Two constructs, CVC and VCV, were incubated in the tris-buffered saline buffer at +4 °C for about 6 months and analyzed by SEC. *A*, construct CVC revealed an appearance of a hexamer peak at ∼12.6 ml, which accounts for 25% of total protein. The CVC hexamer demonstrated stable assembly in the presence (*B*) or absence (*C*) of chloride. *B*, CVC peaks from (*A*) were pooled and reanalyzed in tris-buffered saline (with chloride). *C*, CVC peaks from (*A*) were pooled and reanalyzed in 100 mM sodium phosphate, pH 7.5 (no chloride). SEC, size-exclusion chromatography.

Ambiguity in the chain composition of NC1 trimers assembled from monomers precluded further studies of the *in vitro* assembly of the NC1 hexamer. To overcome this problem, we recombinantly produced FLAG-tagged single-chain NC1 trimers with specific hetero-compositions CVC and VCV using the same design as in transgenic analysis but without a fluorescent protein (Fig. S16). Although transgenic expression data revealed the biological activity of the CVC composition (Fig. 5), we proceeded with both variants of heterotrimers for comparison reasons. Trimers were expressed and purified using an anti-FLAG resin (Fig. S17) at high Cl^-^ concentration (150 mM), concentrated to at least 1 mg/ml protein concentration, and incubated at 37 °C overnight, following an established protocol for efficient assembly of mammalian NC1 hexamers starting from monomers (26) or single-chain trimers (28). Surprisingly, neither CVC nor VCV demonstrated prominent hexamer peaks but trimer peaks (technically “monomer” for a single-chain trimer) on the SEC. The trimer peaks of CVC and VCV were individually pooled, concentrated to ∼10 mg/ml, incubated at 4 °C for a month, and reanalyzed on the SEC. In this case, CVC was found to form ∼25% of a hexamer peak, whereas VCV did not show any detectable amount of the hexamer (Fig. 6*A*). CVC trimer and hexamer peaks were individually pooled and rerun on the SEC without or with Cl^-^ depletion (Fig. 6, *B* and *C*). Surprisingly, no dissociation of the hexamer into trimer after Cl^-^ depletion was observed (Fig. 6*C*) unlike the case with mammalian NC1 hexamer (28, 52). Thus, the CVC composition of NC1 was able to spontaneously form a hexamer although this process was highly inefficient. Moreover, once assembled the CVC hexamer was not dependent on chloride pressure, thus confirming the Cl^-^ independent mechanism of stability (30).

### Biophysical characterization of NC1 domains

**Figure 7:**
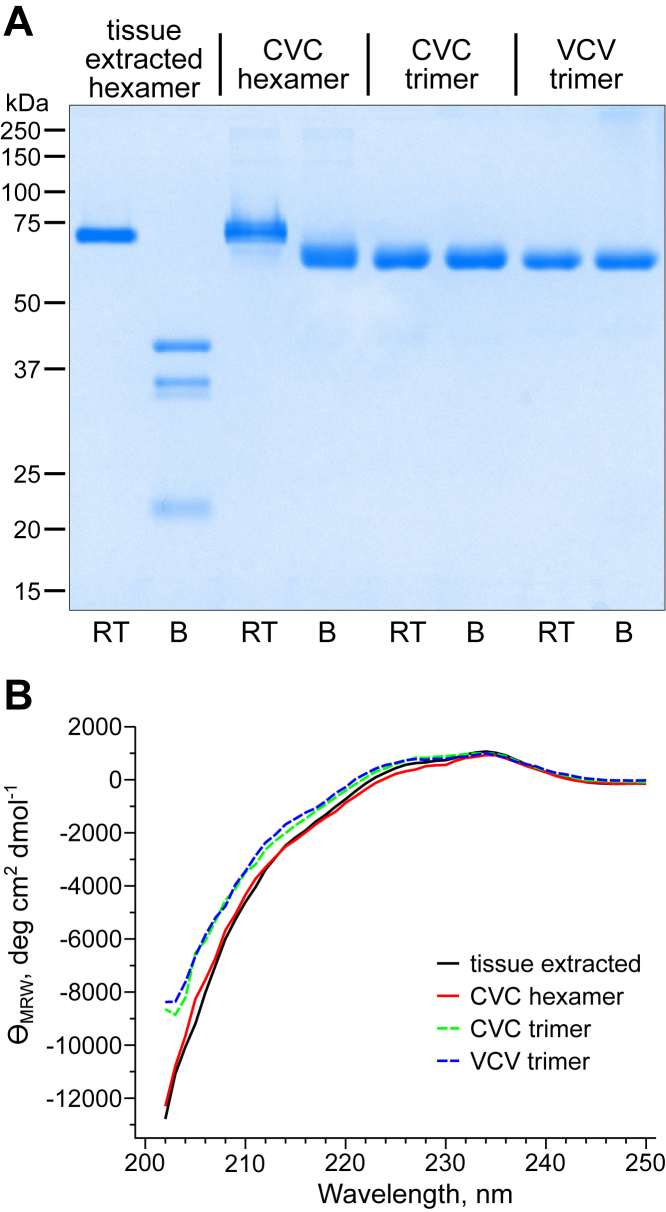
**Characterization of tissue-extracted and recombinantly produced NC1 domains**. *A*, *Drosophila* hexamers revealed SDS resistance. Tissue-extracted NC1 hexamer, CVC hexamer, CVC trimer, and VCV trimer were analyzed on the SDS-PAGE without (RT, room temperature) and after sample boiling (*B*). The tissue-extracted and recombinant CVC hexamers demonstrated SDS resistance when samples were not boiled. The samples were run on a regular 12% SDS-PAGE and Coomassie stained. *B*, the secondary structure of tissue-extracted and recombinant hexamers differs from trimers as revealed by CD spectra. All variants of trimers and hexamers of *Drosophila* NC1 have similar spectra. Nevertheless, tissue-extracted and recombinant CVC hexamers demonstrate more negative signal below 220 nm, indicating some secondary structure rearrangement upon hexamerization. NC1, noncollagenous domain 1.

We compared the tissue-extracted hexamer with recombinantly produced CVC and VCV. The SDS-PAGE analysis revealed the presence of dimer and monomer bands as previously reported (23, 24) (Fig. 7*A*) for the tissue-extracted hexamer. Surprisingly, a protein sample prepared at room temperature without boiling ran as a single band with an apparent mass higher than dimeric bands (Fig. 7*A*), a feature never observed for mammalian species analyzed in our lab (*i.e.*, human, mouse, and bovine, see (30)). Thus, the *Drosophila* NC1 hexamer is SDS-resistant in a regular SDS-PAGE, which was later used for a gel-based hexamer assembly assay (*vide infra*). Note that nonboiled CVC hexamer migrates at the same position as a nonboiled tissue-extracted hexamer.

The far UV CD spectrum of the *Drosophila* NC1 hexamer demonstrated a similar to mammalian shape (16), though with a unique signature of a split maximum peak at ∼225 and ∼235 nm (Fig. 7*B*). The CD spectroscopy showed a structural difference between trimers and hexamers (Fig. 7*B*). Indeed, CVC and VCV trimers have almost identical spectra, whereas CVC hexamer and tissue-extracted NC1 hexamer formed a different group. Thus, the assembly of a hexamer is accompanied by changes in the secondary structure content. A slight difference between CVC hexamer and tissue-extracted hexamer might be due to extra linkers and FLAG-tag sequences in CVC and sulfilimine cross-links in tissue-extracted hexamer.

Overall, the recombinant hexamer is structurally and biophysically similar to the tissue-extracted one.

### Divalent cations trigger the assembly of the hexamer

Analysis of tissue-extracted NC1 hexamer, CVC hexamer, and CVC trimer on SDS-PAGE revealed a difference in their motility when samples were not boiled (Fig. 7*A* and JBC-D-23-01221). This method provided a very simple, robust, and sensitive way (sub microgram amount was sufficient for detection using Coomassie staining) for detection and quantitation of CVC trimer and hexamer in multiple samples instead of using time and material consuming SEC (26, 28, 52) or other methods. We used this new approach to test how different chemicals influence the hexamer assembly.

**Figure 8:**
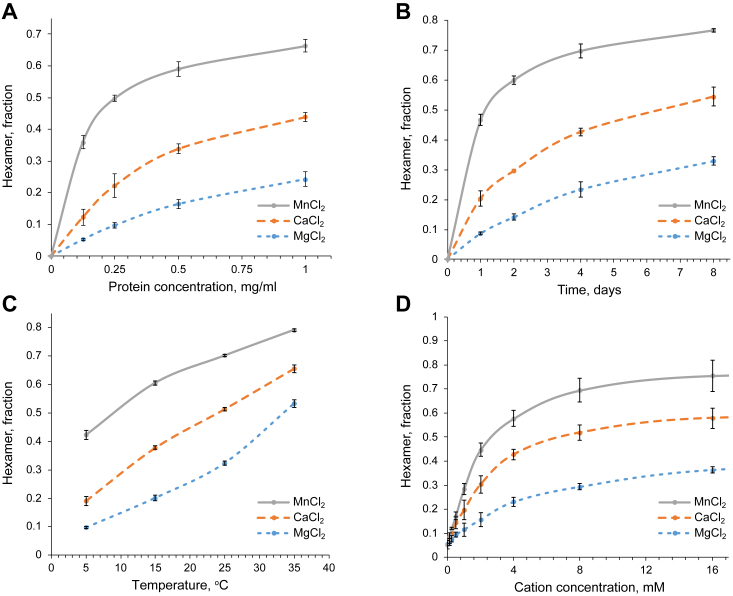
**Hexamer assembly under various conditions.** Protein concentration, time, temperature, and divalent cation concentration accelerate the assembly of hexamer. Manganese promotes the assembly more efficiently than calcium or magnesium. CVC trimer in 25 mM Tris-acetate buffer supplemented with 100 mM Na-acetate was used for hexamer assembly assays. *A*, variation of protein concentrations. Protein at concentrations 0.125, 0.25. 0.5, and 1 mg/ml was subjected to assembly in the presence of 20 mM MgCl_2_, CaCl_2_, or MnCl_2_. at 25 °C for 5 days. *B*, kinetics of assembly. Protein at 1 mg/ml concentration was subjected to assembly in the presence of 20 mM MgCl_2_, CaCl_2_, or MnCl_2_. at 25 °C for 1, 2, 4, and 8 days. *C*, effect of temperature. Protein at 1 mg/ml concentration was subjected to assembly in the presence of 20 mM MgCl_2_, CaCl_2_, or MnCl_2_. at 5, 15, 25, and 35 °C for 7 days. *D*, effect of divalent cation concentration. Protein at 1 mg/ml concentration was subjected to assembly in the presence of 0.125, 0.25, 0.5, 1, 2, 4, 8, 16 mM MgCl_2_, CaCl_2_, or MnCl_2_. at 25 °C for 7 days. Each point was measured three times in independent assembly experiments.

After screening 96 small molecules from the Additive Screen (Hampton Research), we found that certain divalent cations efficiently induced the formation of the CVC hexamer (Fig. S18). Among these Ca^2+^, Mg^2+^, and Mn^2+^ are physiologically relevant ones. We performed a series of assembly experiments in the presence of these divalent cations under various conditions (Figs. 8 and S19). We observed a positive effect of protein concentration, temperature, and cation concentration on the hexamer assembly. In all cases, Mn^2+^ were the most and Mg^2+^ the least efficient ions. We also observed that the presence of Cl^-^ has minimal if any impact on the assembly (Fig. S20).

Although we found Mn^2+^ to be the most efficient cation, due to its extremely low physiological concentration, Ca^2+^ and Mg^2+^ are tentatively prevailing endogenous factors inducing the formation of the NC1 hexamer in *Drosophila*.

Collectively, divalent cations are required for the assembly of the *Drosophila* NC1 hexamer.

### Crystal structures of tissue-extracted and recombinant NC1 hexamers

**Figure 9:**
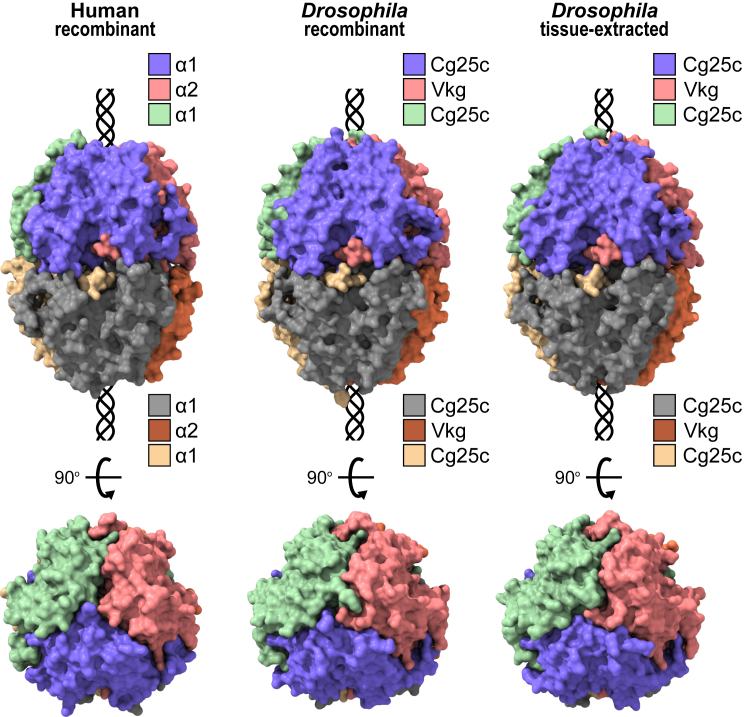
**Crystal structures of *Drosophila* tissue-extracted and recombinant NC1 hexamers and their comparison with human.** Overall structures are similar. Shown are the surfaces of the crystal structures generated and colored individually for each chain. A schematic triple helix is added for orientation purposes. NC1, noncollagenous domain 1.

We attempted crystallization of tissue-extracted NC1 hexamer, recombinantly produced CVC trimer and hexamer, and recombinant VCV trimer. We were able to obtain diffracting crystals of tissue-extracted NC1 hexamer (at 2.98 Å resolution) and CVC trimer (at 1.75 Å resolution) and solved their atomic structures (Fig. 9 and Table S3). The numbering of residues is given for the NC1 domain and does not correspond to the numbering of full-length sequences.

The overall fold of the NC1 monomer is similar to the mammalian one (Fig. 9). Each NC1 monomer contains two C4 subunits, and every C4 subunit is stabilized by three disulfide bonds like in mammals (Fig. S21). Three monomers form a trimer through the domain swapping mechanism previously described (54, 55) but also with unique interactions involving residues different from mammalian sequences (Fig. S22 and Appendix). Those interactions could account for the formation of transiently but stable trimers in *Drosophila* (*vide supra*, Figs. 3 and S9) as opposed to mammalian species. The hexamer architecture appears to be conserved as there are no noticeable shifts in the relative positioning of two trimers (Fig. 9). Indeed, RMSDs for superimposed Cα backbones of human recombinant (PDB: 6mpx) onto *Drosophila* recombinant structure is only 0.77 Å for 1232 pruned atom pairs and 1.19 Å across all 1323 pairs. RMSD of superimposed recombinant and tissue-extracted structures of *Drosophila* NC1 hexamer is 0.43 Å between 1346 pruned atom pairs (across all 1351 pairs: 0.47 Å).

The crystal structure of the *Drosophila* NC1 domain helps to resolve the chain clustering ambiguity (*vide supra*). Analysis of the chain geometries revealed three unique regions, which unambiguously assign Cg25c to the α2-like cluster and Vkg to the α1-like cluster (Fig. S23*A*). Sequence comparison of these three regions (Fig. S23*B*) provides a simpler rule for chain clustering in other animals.

Analogously to human crystal structures of α112 and α345 NC1 hexamers (28, 29, 53), we found multiple PEG molecules associated on the surface and in the inner cavity of the *Drosophila* NC1 hexamer (Fig. S24). Many of them are in contact with more than one chain. Such molecules suggest the existence of natural ligands that could assist in the trimer or hexamer assembly and stability *in vivo*. They also provide a rationale for the design of pharmacological chaperones (56).

Distribution of side chains on a surface though significantly differs from mammals and forms a unique map of electrostatic potential (Fig. S25). Thus, despite the conservation of the overall fold of the NC1 hexamer, the surface evolved into its entity.

### Loss of chloride ions and gain of divalent cations at the hexamer interface

The surface topology and electrostatic potential of a trimer interface for the hexamer formation are also different from mammals, which points to a unique mechanism of hexamer assembly (Fig. S26).

**Figure 10:**
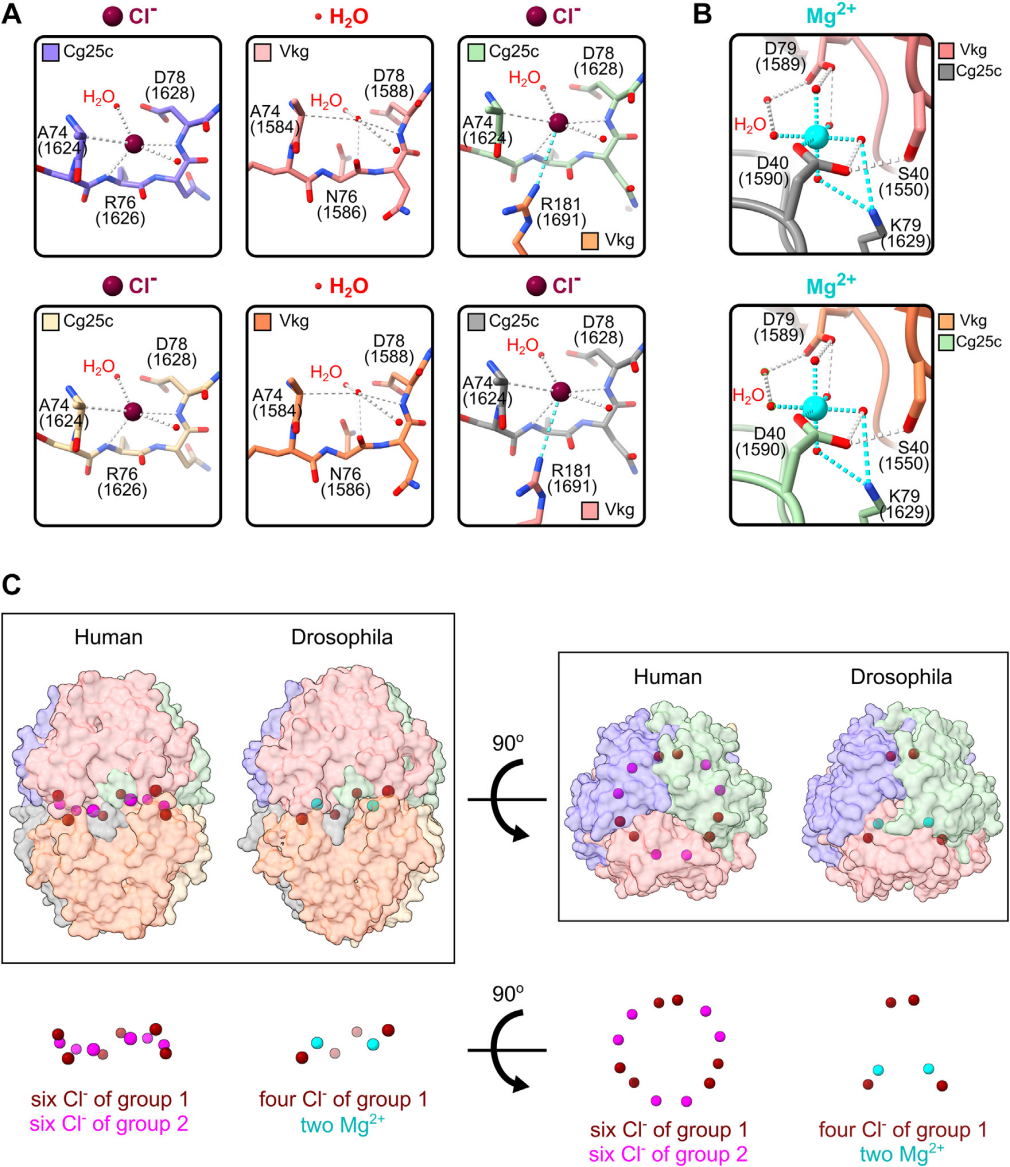
**Ions at the NC1 hexamer interface.***Drosophila* revealed a unique set of ions at the hexamer interface. Unlike in reported mammalian structures stabilized by 12 chloride ions, *Drosophila* has four identical chloride ions and two unique divalent cations. The numbering of residues is given for the NC1 domain and for the full-length sequences in *brackets*. *A*, chloride ions. Only Cg25c chains coordinate Cl^-^ ions, whereas similar regions in Vkg chains coordinate water molecules instead due to the different geometry of the backbone (the N76(1586) carboxyl group of Vkg coordinates a water molecule, whereas the R76(1626) amino group of Cg25c coordinates a chloride ion). Note that only two of four Cg25c chains coordinate chlorides that stabilize the hexamer interface *via* ionic interaction with the side chain of R181(1691) from Vkg, which belongs to an opposite trimer. Distances are reported in Table S4. *B*, Mg^2+^ ions. Two NC1 monomers from opposite trimers coordinate Mg^2+^ ions *via* a series of ionic and hydrogen bonding, which involves multiple water molecules. Magnesium coordination has an octahedral molecular geometry. Ionic interactions are shown with *cyan dash lines*, whereas hydrogen bonds with *white dash lines*. Distances are reported in Table S5. *C*, overall ion positioning in human and *Drosophila* structures. *Drosophila* NC1 hexamer has evolved to have a modified set of ions at the trimer–trimer interface. *Drosophila* preserved four out of six chloride ions of group 1 (sitting deeper in the trimer and not accessible for dynamic exchange with solvent). All six chloride ions of group 2 are missing in *Drosophila*, but two Mg^2+^ cations were found instead. Those Mg^2+^ are not readily accessible for dynamic exchange either.

Out of 12 chloride ions found at the hexamer interface in mammals (28, 29, 53, 57) only four were found in *Drosophila* (Fig. 10*A* and Table S4). All four belong to group 1 ions (28) and each one is coordinated by a loop of residues A74-D78(Cg25c:1624–1628/Vkg:1584–1588). Only Cg25c chains were found to contain chloride ions, while Vkg chains contain water molecules instead. As in mammals, these chloride ions are mainly coordinated by the main-chain atoms. The geometry of the A74-D78(1584–1588) region in Vkg chains is incompatible with the chloride ion coordination (Fig. 10*A*). In mammals all six chains contain chloride ions in the analogous loops. Moreover, out of four chlorides, only two are involved in interaction with Vkg R181(1691) from the opposite trimer (Cg25c has T180(1730) instead of R and cannot interact with the other two Cl^-^), thus the role of group 1 chloride ions in stabilizing the hexamer structure is significantly compromised in *Drosophila*.

In mammals, it is group 2 (consisting of other six ions) that contributes most to the hexamer assembly and stability. None of group 2 chlorides were found in *Drosophila*. Instead, two magnesium ions were found at the trimer–trimer interface (Fig. 10*B* and Table S5). Single D40(1590) of Cg25c directly forms a salt bridge with Mg^2+^, while K79(1629) of the same Cg25c chain and D79(1589) of the Vkg chain interact with water molecules clustered around the divalent cation. Also, S40(1550) of the same Vkg chain forms a hydrogen bond with D40(1590) of Cg25c. Altogether, these interactions stabilize the trimer–trimer association as these Cg25c and Vkg chains belong to opposite trimers. As the Mg^2+^ coordination involves Cg25c and Vkg chains, two other Cg25c chains can not coordinate two more Mg^2+^ ions as they oppose other Cg25c but not Vkg chains. In this case, Cg25c cannot provide specific residues for binding. The requirement to have Cg25c and Vkg for coordination of the magnesium ion predetermines the only possible positioning of two trimers in the hexamer.

Collectively, unlike in mammals *Drosophila* NC1 hexamer interface is stabilized by four Cl^-^ and two Mg^2+^ ions (Fig. 10*C*).

### The *Drosophila* hexamerization model is unique to Diptera

**Figure 11:**
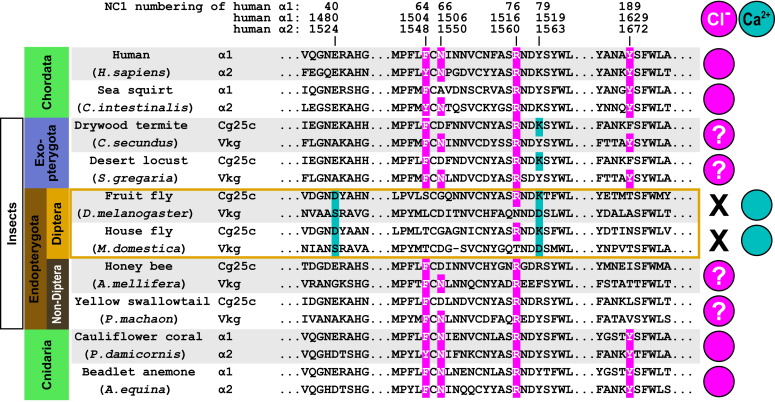
**Diptera has a unique NC1 hexamer interface.** Conservation of residues coordinating the divalent cations in *Drosophila* is restricted to the Diptera genus. The numbering of residues is given for the NC1 domain and the full-length human sequences. Sequence alignment of representative animals reveals conservation of Ca^2+^/Mg^2+^ coordinating residues at positions 40 (human α1:1480/α2:1524) and 79 (α1:1519/α2:1563) only in Diptera (highlighted with *cyan background*). Residues coordinating Cl^-^ ions of group 2 at positions 64 (α1:1504/α2:1548), 66 (α1:1506/α2:1550), 76 (α1:1516/α2:1560), and 189 (α1:1629/α2:1672) are highlighted with *magenta background*. Diptera largely lacks such residues.

We performed an analysis of residues coordinating group 2 Cl^-^ in mammals and Mg^2+^ in *Drosophila* in sequences of representative animals. We found that a full set of Mg^2+^ coordinating residues is only conserved in Diptera. (Fig. 11). Thus, the divalent cation-driven mechanism of assembly and stability appears to be unique to Diptera. The role of group 2 Cl^-^ ions remains unknown for Exopterygota and other non-Diptera Endopterygota.

## Discussion

*Drosophila* collagen IV shares a common domain organization with humans, which includes the C-terminal NC1 domain, the collagenous central part, the 7S-like region at the N terminus, and a pair of cysteines (45) which correspond to those forming a cystine knot within the CB3 fragment of human collagen IV (58). Despite the similarities, there are own traits which include a unique distribution of triple helical segments and interruptions, a unique set of unpaired cysteines within the collagenous domain of Cg25c, a missing cysteine and a flipped site for N-glycosylation in 7S, a missing cysteine loop (found in mammalian α2 chain), and an extension or “tail” after the NC1 domain in the Vkg chain (Fig. 2 and S2). While commonality between evolutionarily distant organisms helps to identify the fundamental principles of this biomolecule, variability points to adaptive flexibility and functional potential. Those sites of variabilities are hot spots for evolutional adaptation and the invention of new functions. Overall conservation of collagen IV in *Drosophila* preserves its fundamental function to build a structural scaffold for the BM, while variabilities of the collagenous domain and the NC1 domain surface are tools for broadening functional space from structural to an actively communicating scaffold. Here, we explored the structure and assembly of the *Drosophila* NC1 domain.

Clustering of vertebrate NC1 sequences into α1- and α2-like groups was not straightforward for *Drosophila* sequences. With some ambiguity, Cg25c and Vkg NC1 sequences could be assigned to α2- and α1-like clusters respectively. If followed by a mammalian rule of combining two odd-numbered and one even chains that would lead to a false prediction of a protomer composition as two Vkg and single Cg25c chains. *Drosophila* collagen IV composition is thus an exception. Extra care should be taken when analyzing the composition of collagen IV protomers in animals other than mammals. *A priory* even homotrimeric compositions cannot be excluded.

Isolation of endogenous NC1 hexamer from *Drosophila* pellet showed the presence of both Cg25c and Vkg chains (Fig. S7). It suggested a heterotrimeric composition, but copurification of two different homohexamers or heterohexamers composed of two different homotrimers was not excluded. However, the latter possibility could be excluded as it would contradict the discovery of a sulfilimine bond between two sequences of Cg25c (23), where the bond links two NC1 monomers from opposite trimers. The heterotrimeric composition was supported by recombinant expression of NC1 monomers, where we detected transient heterotrimers. Transgenic experiments clarified that of all four possible variants of NC1 trimer including homo and heterotrimers only heterotrimer Cg25c:Vkg:Cg25c (CVC for short) is deposited into the BM.

While tissue-extracted NC1 domain was found to be a *hexamer* and the CVC heterotrimer got deposited to the BM in the transgenic experiments *via* presumably hexamerization process with endogenous NC1 trimers, our recombinant trimers failed to assemble into hexamers *in vitro* under conditions established for mammalian samples, that is, at high chloride concentration. Surprisingly, though, after extended storage at 4 °C, the CVC sample revealed the formation of a hexamer. The spontaneously assembled CVC hexamer showed no requirement of chloride in solution (chloride “pressure”) to maintain its hexamer integrity. Removal of chloride from solution did not cause dissociation of the hexamer (Fig. 6*C*) as was the case for mammalian collagen IV (26, 28, 52) This is in accord with the observation that a tissue-extracted NC1 hexamer from *Drosophila* reveals an SDS resistance regardless of chloride presence in solution (30). Chloride pressure is a primordial invention for the assembly and stability of collagen IV hexamers found in Cnidaria to mammals but with exceptions like *Drosophila* (30).

Formation of the NC1 hexamer is accompanied by a small yet detectable change of the secondary structure as revealed by the circular dichroism spectra of recombinant single-chain trimers and the CVC or tissue-extracted hexamers. Yet, no atomic structures exist of any NC1 trimer to understand this transition.

Screening of multiple small molecules as potential triggers of the hexamer assembly in *Drosophila* revealed multiple divalent cations trigger assembly of the hexamer. Among them, Mg^2+^, Ca^2+^, and Mn^2+^ are biologically relevant candidates for promoting assembly *in vivo*, with Mg^2+^ being the least and Mn^2+^ the most efficient trigger. We observed similar trends (as in the case of human chains) in the assembly of the *Drosophila* NC1 hexamer, with the positive impact of protein concentration, time, temperature, and trigger concentration, although overall kinetics was several times slower (28). Indeed, under similar protein concentration, temperature, and saturating trigger concentration *Drosophila* assembly takes days instead of overnight incubation. Given the lower physiological temperature and short life cycle in *Drosophila* such slow assembly is physiologically irrelevant. Other factors accelerating assembly thus should exist. They could be other small molecules, macromolecules, or assistance from cell receptors. Indeed, integrins were shown to facilitate collagen IV deposition in *Caenorhabditis elegans* (59), and integrins and syndecan act as collagen IV receptors in *Drosophila* (60, 61), whereas heparin which mimics heparan sulfate chains of multiple BM proteins is known to bind the NC1 domain and the collagenous domain of mammalian collagen IV (60, 62, 63). Interactions with nidogen and perlecan associate collagen IV protomers with the laminin network (10, 64) and hence promote selective binding to the BM.

We solved the crystal structures of tissue-extracted and recombinant NC1 hexamers and found that the tissue-extracted complex is indeed a heterotrimer of the same composition as the recombinant CVC single-chain construct. The overall fold of *Drosophila* hexamer is the same as in mammals, which highlights the primordial structure of the NC1 domain. Nevertheless, the trimer-to-trimer interface of the hexamer as well as the surface of the hexamer is different. In mammal structures, there is a ring of 12 chloride ions at the interface, which structurally belong to two different groups, six ions in each. At the hexamer interface of *Drosophila* there are no chloride ions of group 2 and an incomplete set of ions of group 1 (Fig. 10*C*). Instead of group 2 chloride ions, we found two magnesium cations bonding two trimers together. The magnesium ions are located deep enough from the solvent and do not seem to be able to exchange with the solution environment as is the case for group 2 chloride ions in mammals (28, 29, 52). Indeed, the removal of divalent cations from the solution does not cause dissociation of the assembled hexamer. The presence of chloride in solution during the hexamer assembly has a positive but tiny effect on the complex formation. The partial presence of group 1 chloride ions seems to be an evolutional “memory” of a primordial mechanism though still with some positive impact on the assembly. The absence of group 2 chloride ions in *Drosophila* emphasizes their role in maintaining the hexamer structural and conformational integrity in mammals (28, 52). Why has *Drosophila* lost group 2 chlorides and invented a new mechanism for the hexamer assembly?

The mammalian NC1 domain is sensitive to Cl^-^ concentration, that is, it remains monomeric at low (<30 mM) and undergoes assembly into hexamers at high (>70–100 mM) Cl^-^ concentration (26). When Cl^-^ is depleted the NC1 hexamer (unless it is crosslinked by covalent sulfilimine bonds) dissociates back into monomers (26). Does *Drosophila* extracellular space provide sufficient chloride concentration for the same mechanism? We assume that it should be close to that in the circulating fluid termed hemolymph, which washes around organs and tissues. For insect hemolymph, it has been pointed out many times that sodium and chloride are usually low in percentage; while potassium, phosphorus, calcium, and magnesium are present in relatively high concentrations, as reviewed in (65). More systematic analysis revealed quite a wide range of chloride concentrations in the hemolymph of different insects, ranging from 7 mM in a silk moth to 80 mM in a dragonfly (66). It was suggested later that a relatively high concentration of chloride is characteristic of the Exopterygota, whereas a low proportion is characteristic of the Endopterygota (where *Drosophila* belongs) (67). Indeed, the hemolymph of *Drosophila* was also found to have a low chloride concentration of 36 mM (68). It closely matches chloride concentrations in hemolymph of several species from Hymenoptera order found in a range of 37 to 41 mM (69). Such concentrations would be insufficient to drive the hexamer assembly in mammals, so the primordial chloride-dependent mechanism should be compromised in *Drosophila* (and possibly also in the Endopterygota group of insects) and a new mechanism had evolved that relies on divalent cations.

Although manganese is an essential element for maintaining life also in insects (70) its physiological concentrations are submicromolar at which the NC1 assembly would be negligible. On the other hand, Ca^2+^ concentration in *Drosophila* hemolymph was reported to be 0.5 mM in adults (71) and 9 mM in third instar larvae (68). In the hemolymph of other insects, Ca^2+^ varies from 2.7 to 10 mM and Mg^2+^ from 1.4 to 22 mM (72). So, both ions can contribute to the NC1 assembly. Since most of the Ca^2+^ and Mg^2+^ measurements were done for a total amount, which also included bound forms of divalent cations, the amount of soluble forms of calcium and magnesium should be less. In any case, the overall kinetics of assembly even at the highest possible Ca^2+^ or Mg^2+^ concentrations (close to 20 mM) should still be unacceptably slow as discussed above. It again reemphasizes the requirement for either additional small molecule ligands, specialized “helper” macromolecules assisting the assembly, involvement of cell receptors in prepositioning collagen IV molecules, or combinations of these factors. The existence of those other factors would also provide a mechanism that prevents premature collagen IV scaffold assembly inside the cell. However, an alternative possibility exists where the “tail” sequence of Vkg NC1 makes the protomer to be a latent form.

Unlike in vertebrates, one chain of fruit fly collagen IV, Vkg, contains an additional sequence (“tail”), following the NC1 domain with yet unknown function. Even within the same genus of *Drosophila*, NC1 sequences of Vkg have very little conservation, which is restricted to ∼25 C-terminal residues. Given high variability of the “tail” sequences and poorly structured model prediction in the AlphaFold Protein Structure Database (73, 74), this presumably flexible hanging around polypeptide must be prone to proteolytic cleavage and removal somewhat similar to unstructured regions between telopeptide and the C-propeptide in fibrillar collagens (75, 76) and a hinge region between the trimerization domain and endostatin in multiplexins (collagens XV and XVIII) (77, 78). We performed several attempts to model a “tail” structure in the context of the NC1 trimer using the AlphaFold multimer approach (73, 74). An ensemble of predicted-with-low-confidence structures demonstrated sufficient length of the “tail” to rich the interface for hexamer assembly and in about every other case the C-terminal residues were predicted to interact with that interface. A possibility exists that the Vkg “tail” could function as an assembly blocker to prevent premature formation of the hexamer inside the cell or in hemolymph on the way to the site of active BM growth or remodeling making the protomer a latent form. On-site cleavage of the “tail” would trigger the assembly. Such tails were not reported in vertebrate chains though. Moreover, in humans and mice, a mutation causing a short extension of the NC1 domain leads to pathologies (49). So, nonconventional “tails” could be harmful as they interfere with the surface of NC1.

In our previous study, a *Drosophila* transgene with R76A(Cg25c:R1626A) NC1 mutation resulted in the mutant protein being incorporated into BMs *in vivo* (26). Residue 76 is noncritical for NC1 trimerization to form protomers but was expected to compromise the assembly of the hexamer as each R76(α1:1516/α2:1560) forms double salt bridges with E175(1615) of α1 or corresponding E173(1657) of α2 in mammalian structures (26). While in mammalian NC1 hexamer, there are six such pairs observed, in *Drosophila* only four are possible as the Vkg chain contains N76(1586) instead. In the solved crystal structures, we indeed observed four pairs of double salt bridges from R76(1626) of each Cg25c to either E173(1723) of Cg25c or E175(1685) of Vkg. Thus, the overall importance of R76 in stabilizing the hexamer in *Drosophila* is less significant than in mammals. Moreover, the divalent cation stabilization of the hexamer could attenuate detrimental effects or R76A mutation.

The divalent cation mechanism of the NC1 hexamer assembly discovered in *Drosophila* seems to be an adaptation to a low chloride concentration in the extracellular space. How universal is it for other insects, that is, Endopterygota for which low chloride concentration in hemolymph was reported? Sequence alignment of other Endopterygota species showed that the *Drosophila* model is unique to the order Diptera (Fig. 11). Holometabola (or Endopterygota) are, given their evolutionary age, by far the most species-rich subgroup of insects and comprise more than 60% of all described metazoan species (79). Such richness could be attributed to enormous anatomical and physiological diversity, among which the collagen IV assembly mechanism. Endopterygota insects other than Diptera must have evolved different mechanisms of collagen IV hexamerization.

Other than having a different mechanism of the hexamer assembly, the NC1 domain in *Drosophila* evolved to have a different map of solvent-accessible side chains, which makes the surface functionally unique. Despite the conservation of the overall fold of NC1, the surface seems amenable to significant changes without compromising the fold. Thus, the surface has a capacity for evolutional plasticity and functional adaptation. The NC1 surface modulates chain selection during trimeric protomer assembly, determines a mechanism of the hexamer assembly, and possibly defines other functions like building superstructures by interacting along the collagenous domain of other collagen IV molecules in a very regular pattern (80, 81), binding to integrins (82, 83), bone morphogenic proteins (84, 85), heparin (62, 63), and functions yet to be discovered for this primordial domain.

**Figure 12:**
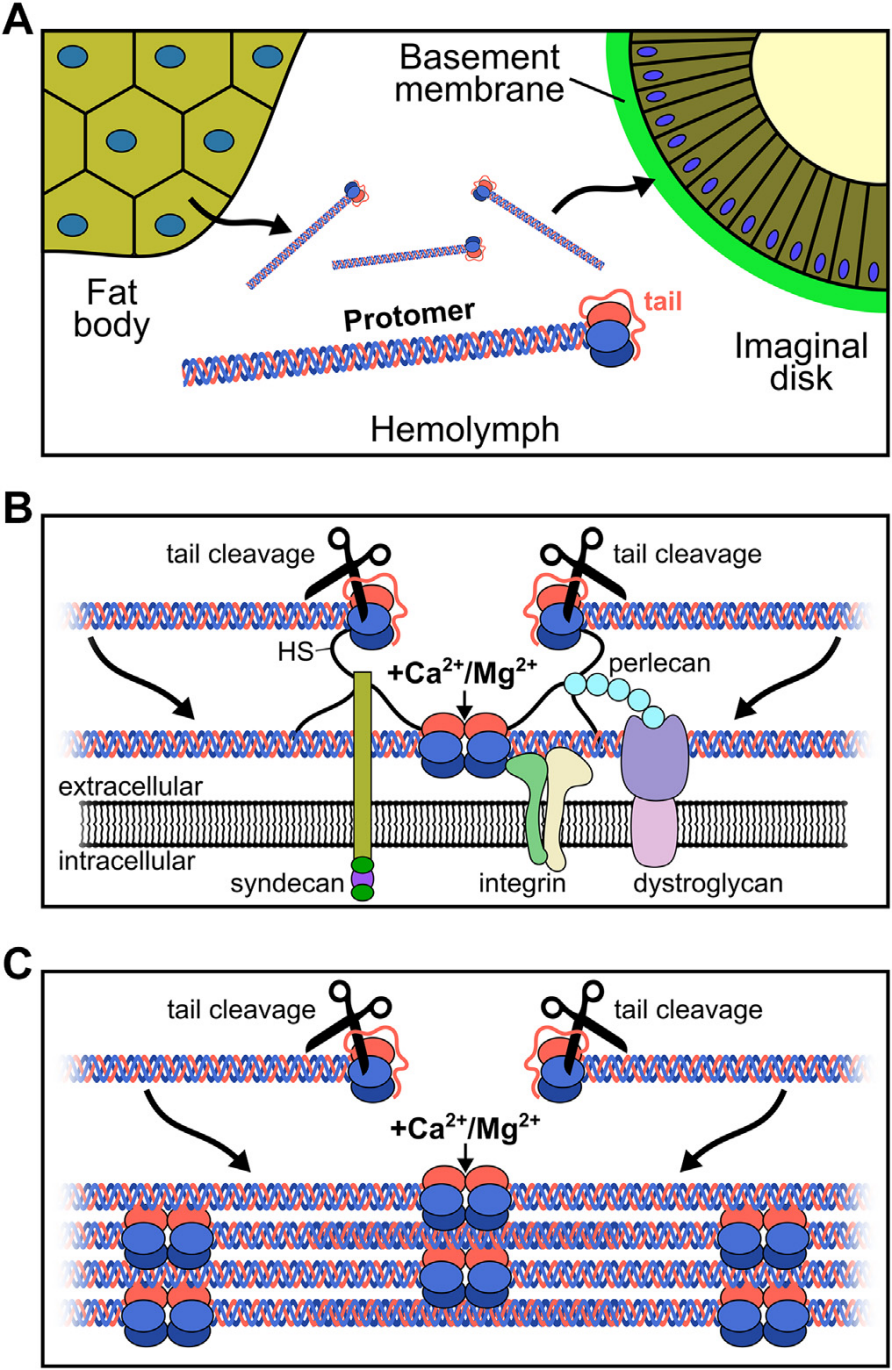
**Collagen IV scaffold assembly in *Drosophila*.***A*, assembled protomers of collagen IV are secreted to hemolymph and delivered to sites of assembly. The Vkg tail is presumably protecting protomers from premature assembly. Once delivered the tail is cleaved off to expose the NC1 hexamer interface for assembly. *B*, primary assembly of the protomers on the cell surface is putatively facilitated by interactions of cell receptors and other macromolecules with the NC1 domain. *C*, continuous assembly of the collagen IV scaffold is also facilitated by lateral interactions with the assembled molecules. The NC1 hexamer assembly requires the presence of the divalent cations. HS, heparan sulfate; NC1, noncollagenous domain 1.

Unlike vertebrates, *Drosophila* is one of the animals where secretion of the protomers from cells and assembly of the collagen IV scaffold was shown to take place at separate sites (61). A mechanism, yet unknown, of distant and specific delivery of these molecules for deposition (Fig. 12) can provide clues for developing collagen IV protein replacement therapy in such diseases as Gould (86) and Alport syndromes (87). Loss of collagen IV in Alport syndrome causes kidney failure and there is no curative treatment available (88). In our transgenic experiments, we found that the NC1 trimer gets incorporated into the BM. Although it is only one-seventh part of the whole collagen IV molecule, the NC1 domain bears information for homing it to the BM. It provides an initial clue of using NC1-containing fragments of collagen IV for developing protein replacement therapy in humans (89).

In conclusion, we explored the composition, structure, and assembly mechanism of the NC1 hexamer in *Drosophila*, which established a new venue for studying the evolution and function of collagen IV. As an exception to the rule, the *Drosophila* example emphasized the role of chloride pressure and built-in ions in the assembly and maintenance of the human NC1 hexamer. It pointed unambiguously to group 2 chloride ions that dynamically stabilize the hexamer structure and prevent the appearance of the autoimmune epitopes in Goodpasture disease. Moreover, selective incorporation of the *Drosophila* NC1 trimer into the basement membrane gives hope for developing protein replacement therapies.

## Experimental procedures

### Accession numbers of sequences and numbering of residues

The following collagen IV sequences from UniProt database were used for the analysis and numbering of chains: human α1 chain (P02462, isoform 1), human α2 chain (P08572), human α3 chain (Q01955, isoform 1), human α4 chain (P53420), human α5 chain (P29400, isoform 1), human α6 (Q14031, isoform 1), *Drosophila* Cg25c chain (P08120), and *Drosophila* Vkg chain (Q9VMV5).

The NC1 domain numbering starts at residues 1441 for α1, 1485 for α2, 1441 for α3, 1461 for α4, 1457 for α5, 1463 for α6, 1551 for Cg25c, and 1511 for Vkg.

### Purification of NC1 hexamer from *Drosophila* pellet

A pellet from the production of fly extract, which contained most of the insoluble extracellular matrix, was purchased from *Drosophila* Genomics Resource Center, Indiana University (Fly Pellet, DGRC Stock 1661280; https://dgrc.bio.indiana.edu//stock/1661280). Initial extraction and purification of the NC1 hexamer were performed as described (90). Pooled fractions corresponding to the hexamer peak from the SEC were dialyzed against 20 mM Tris–HCl, pH 8, and run over the Q-sepharose column (prepacked 5-ml column from Cytiva) equilibrated against the same buffer. The NC1 hexamer was eluted with a linear NaCl gradient from 0 to 1 M at around 250 mM. The major peak fractions were pooled, dialyzed against 5 mM sodium phosphate buffer pH 7.5, and ran over a 2-ml hydroxyapatite column (packed with Ceramic Hydroxyapatite Type II Media, Bio-Rad) to bind other impurities. The NC1 hexamer was found in the flow-through fraction. The final yield was ∼1 mg starting from ∼100 g of wet pellet.

### Mass spectrometry analysis

Tissue-extracted and purified NC1 hexamer candidate was run on a regular 12% SDS-PAGE without boiling the samples. A single band was excised and subjected to in-gel tryptic or chymotryptic digestion to recover peptides. The resulting peptides were analyzed by data-dependent liquid chromatography with MS/MS. Briefly, peptides were autosampled onto a 200 mm by 0.1 mm (Jupiter 3 μm, 300A), self-packed analytical column coupled directly to an LTQ linear ion trap mass spectrometer (Thermo Fisher Scientific) using a nanoelectrospray source and resolved using an aqueous to organic gradient. Both the intact masses (MS) and fragmentation patterns (MS/MS) of the peptides were collected in a data-dependent manner, utilizing dynamic exclusion to maximize the depth of coverage. Using SEQUEST (https://proteomicsresource.washington.edu/protocols06/sequest.php) (91), resulting peptide MS/MS spectral data were compared with and scored against predicted tryptic or chymotryptic peptides from a canonical *Drosophila* protein sequence (UniProt) to which common contaminants and reversed versions of each protein were added. Peptide spectral matches were collated, filtered, and compared using Scaffold (Proteome Software). A Fisher’s exact test was performed within Scaffold to evaluate aggregate peptide spectral match differences between Cg25c, Vkg, and the control.

### Cloning of NC1 domains from Cg25c and Viking genes

Plasmids pDONR221_Cg25c and pDONR221_Vkg (92) were used as templates for PCR to amplify gene fragments encoding NC1 domains of *Cg25c* and *Viking* genes respectively. The primers used for amplification are reported in Table S1.

Sequences of individual NC1 domains were PCR amplified using pairs of primers: NC1-Cg25c_fw and NC1-Cg25c_rv for the *Cg25c* gene and NC1-Vkg_fw and NC1-Vkg_rv for the *Viking* gene. The backbone of the pRcX vector was PCR amplified using a pair of oligonucleotides pRcX_fw and pRcX_rv. The amplified DNA fragments were assembled in-frame with the SPARC signal peptide and the FLAG tag of the pRcX vector (93) using the HiFi Assembly Kit (NEB). The resulting plasmids are shown in Table S2.

The NC1 sequences of *Cg25c* and *Viking* were recloned from the pRcX (Flag-tagged) plasmids into the pRcH (His-tagged) vector using restriction sites NheI and BspDI. The resulting plasmids are shown in Table S2.

All generated plasmids were sequence-verified, and their encoding protein sequences are shown in Fig. S8.

### Cloning of NC1 single-chain trimers

For single-chain trimeric constructs, we use a simplified abbreviation, where C stands for *Cg25c* and V for *Viking*. For example, CVC trimer stands for a single polypeptide chain of *Cg25c*, *Viking*, and *Cg25c* NC1 genes. All four possible combinations of single-chain NC1 trimers, that is, CCC, VVV, CVC, and VCV were cloned into pcDNA_mEmerald vector using the HiFi Assembly Kit (NEB). For each construct, three corresponding fragments 1, 2, and 3 were PCR amplified using specific primers (Table S1) and coassembled with the plasmid backbone, which was PCR amplified using mEmer_fw and FLAGrv primers. As a result of the design, a heptapeptide linker GSSASSG was introduced between each pair of *Drosophila* genes to ensure flexibility between domains. Final plasmids (Table S2) encoded the SPARC signal peptide, the FLAG tag, the construct of interest, and the mEmerald fluorescent protein. Expression, secretion, and solubility of each construct were verified using the ExpiCHO Expression System (Gibco). The resulting plasmids are shown in Table S2.

Plasmids encoding single-chain CVC and VCV NC1 trimers without mEmerald (pcDNA-*CVC* and pcDNA-*VCV*) were generated using the mutagenesis kit (NEB). Namely, the sequence encoding mEmerald was omitted by over-the-plasmid PCR amplification from pcDNA_*CVC-mEmerald* and pcDNA_*VCV-mEmerald* plasmids using the primers pcDNAstop_fw and mEm-lnk_rv (Table S1) and subsequent ligation. The resulting plasmids are shown in Table S2.

All generated plasmids were sequence-verified, and their encoding protein sequences are shown in Figs. S11–S14 and S16.

### Transient expression and purification of proteins

We transfected the plasmids into expiCHO-S cells (Gibco) according to the ExpiCHO Expression System User Guide and followed the Max Titer Protocol with two modifications: the second feed was added on day 4 and the final culture was collected on day 8. The cells were pelleted by centrifugation at 4000*g* for 15 min, and media were collected for protein purification.

The media were extensively dialyzed against the tris-buffered saline buffer before affinity purifications. The recombinant proteins fused with an N-terminal FLAG-tag were purified as described (28). The N terminally His-tagged proteins were purified on Ni-NTA resin (Qiagen). SEC using Superdex 200 Increase 10/300GLcolumn (GE Healthcare) was used as a final purification step.

### Expression of collagen IV transgenes in *Drosophila*

For expression of C terminally mEmerald-tagged collagen IV NC1 single-chain trimers CCC, VVV, CVC, and VCV in *Drosophila* larvae, we used the GAL4-UAS binary expression system (94) under control of the Cg-GAL4 driver, expressed in the fat body, the main source of collagen IV for BMs in the larva (61). To generate the corresponding transgenic UAS lines, NC1trimer-mEmerald constructs were recloned into the pValium10-roe vector for transgenic insertion into the attP40 site in chromosome II of *y v sc; P{CaryP}attP40* flies. The genotypes of the lines thus generated were: *y v sc; UAS-CCC-mEemrald*, *y v sc; UAS-VVV-mEemrald*, *y v sc; UAS-CVC-mEemrald* and *y v sc; UAS-VCV-mEemrald.* Crosses of these lines to *w; Cg-GAL4* flies were maintained at 25 °C in standard fly food. Wing imaginal discs and pericardial filter cells were dissected in PBS from third-instar larvae, fixed in 4% paraformaldehyde, and mounted in Vectashield-4′,6-diamidino-2-phenylindole (Vector Laboratories) for imaging in an laser scanning microscope 780 confocal microscope (ZEISS). The mEmerald fluorescence incorporated into the BM was quantified from disc confocal images using ImageJ (https://imagej.net/software/imagej/) and Prism GraphPad (https://www.graphpad.com/). Staining with anti-Cg25c antibody was performed as previously described (95).

### Oligomeric state analysis

SEC was conducted with a Superdex 200 Increase 10/300 Gl gel-filtration column (GE Healthcare), using the ÄKTA FPLC system (GE Healthcare) at a 0.5 ml/min flow rate. Two eluants were used: 25 mM Tris–HCl, pH 7.5, with 150 mM NaCl (tris-buffered saline, Cl^-^-reach buffer), and 25 mM Tris-acetate, pH 7.5, with 150 mM sodium acetate (tris supplemented with Na-acetate, Cl^-^-depleted buffer). Eluting proteins were monitored by *A*_280_. Apparent sizes were calculated using a calibration curve where the logarithm of the molecular mass was plotted against the normalized retention volume of protein standards (Bio-Rad). The area under the hexamer peak was integrated using Unicorn (https://www.cytivalifesciences.com/en/us/shop/chromatography/software/unicorn-7-p-05649) software (GE Healthcare) and expressed as a percentage of the total peak area for quantitation of hexamer assembly.

### CD spectroscopy

Far-UV CD spectra were recorded on a Jasco model J-810 spectrometer equipped with a Peltier temperature control unit (JASCO Corp.) using a quartz cell of 1 mm path length at 20 °C. The spectra were normalized for concentration and path length to obtain the mean molar residue ellipticity.

### Hexamer assembly assay

*In vitro* assembly of NC1 monomers and trimers into hexamers was analyzed by two methods: SEC as previously described (26) and an SDS gel electrophoresis based on the SDS resistance of the hexamer at room temperature. Samples for analysis were prepared using a standard 2× SDS sample buffer at room temperature. When boiled the hexamers dissociated into subunits. A regular SDS-PAGE with Coomassie staining was used for the detection and quantification of hexamer formation. Gels were imaged with the Gel Doc XR+ system (Bio-Rad) and density profiles of individual lanes were measured using the ImageJ v1.53a software (96). Density profiles were further processed with the fityk v1.3.1 program (97) for baseline subtraction and integration of protein bands as a readout of quantity.

### Crystallography and determination of structures

The tissue-extracted NC1 hexamer and recombinant CVC trimer were crystallized in hexagonal forms (space group P6_3_) using the hanging drop vapor diffusion method. The proteins were prepared in 20 mM Tris–HCl, pH7.5 buffer supplemented with 0.15 M NaCl. The tissue-extracted hexamer (∼3.5 mg/ml) was mixed for the drop solution in a 1:1 proportion with a reservoir solution of 35% (+/−)-2-Methyl-2,4-pentanediol, 0.1 M NaCl, 100 mM Hepes pH 7.5. The crystals grew to a final size of 0.15 × 0.15 × 0.1 mm after a couple of weeks at 22 °C. The crystals were directly frozen in liquid nitrogen. The recombinant CVC trimer (∼8.5 mg/ml) was mixed for the drop solution in a 1:1 proportion with a reservoir solution of 0.1 M Na/K phosphate, pH 6.5, 0.2 M sodium chloride, and 26 % (w/v) PEG 1000. The crystals grew to a final size of 0.1 × 0.1 × 0.05 mm after 3 weeks at 22 °C. The crystals were briefly dipped into a cryoprotectant solution containing the reservoir solution and 30% (v/v) PEG 400 and then frozen in liquid nitrogen.

Data collection was performed remotely on crystals cryocooled to 100 K at the Life Sciences Collaborative Access Team beamline 21-ID-G at the Advanced Photon Source, Argonne National Laboratory. Data processing and indexing were performed using iMOSFLM (98) and then scaled and merged using POINTLESS (99). Initial phases were obtained by molecular replacement using AMoRe (100) in CCP4 (101) and the previously solved α1α1α2 NC1 trimer (PDB code: 6MPX) (21) as the search model.

Six chains were found per asymmetric unit in each case. Refinement of the structures was carried out using Phenix (https://phenix-online.org/) (41). The models were manually adjusted between each refinement cycle using Coot (42). Model geometry was assessed using MolProbity (43). The final data collection and refinement statistics are shown in Table S3. For visualization and structure analyses the program UCSF ChimeraX (102, 103) was used.

### Data presentation and analysis

3D images were generated using Blender (www.blender.org). Plots were visualized with the Grace program (http://plasma-gate.weizmann.ac.il/Grace/). Protein structure figures were generated using UCSF ChimeraX (102, 103). Drawings, editing, and labeling of figures were done using the GIMP (www.gimp.org) and Inkscape (inkscape.org) software.

## Data availability

The atomic coordinates and structural factors have been deposited with the Protein Data Bank (PDB codes: 8TXN and 8TYS). The DNA sequences of the constructs are available by request. All other data are contained within the manuscript or Supporting information.

## Supporting information

This article contains supporting information.

## Conflict of interest

The authors declare that they have no conflict of interest with the contents of this article.
