# Supplementary material for: Collagen IV of basement membranes: IV. Adaptive mechanism of collagen IV scaffold assembly in Drosophila
Source: J Biol Chem. 2023 Oct 27;299(12):105394. doi: 10.1016/j.jbc.2023.105394 (PMC10694668; doi:10.1016/j.jbc.2023.105394)
Supplement: Table S1 [file mmc2.docx]

Table S1. **Primers for PCR amplification of DNA fragments.**

| **For cloning individual NC1 domains** | |
| --- | --- |
| NC1-Cg25c_fw | AGGACGACGATGACAAGCTAGCGGGTGAGCCCGGTGCTCCA |
| NC1-Cg25c_rv | CCCGCGGTTAACATCGATTACGAGGAGTTCTTCATGCACACC |
| NC1-Vkg_fw | AGGACGACGATGACAAGCTAGCGGGTGAACCAGCGCCAGCA |
| NC1-Vkg_rv | CCCGCGGTTAACATCGATTAGCTGTTGCCTCGACGACG |
| pRcX_rv | CGCTAGCTTGTCATCGTCGTC |
| pRcX_fw | TAATCGATGTTAACCGCGGGC |
| **For cloning single-chain NC1 trimers fused to mEmerald fluorescent protein** | |
| FLAGrv | CGTACTTGCTAGCTTGTCATCGTCG |
| Cg25c-1_fw | GATGACAAGCTAGCAAGTACGCTGGACTATCTCACCGGTATC |
| Cg25c-1_rv | ACCCGAGCTGGCAGAACTTCCCGAGGAGTTCTTCATGCAC |
| Cg25c-2_fw | GGAAGTTCTGCCAGCTCGGGTCTGGACTATCTCACCGGTATC |
| Cg25c-2_rv | TCCACTAGAAGCGCTGGAGCCCGAGGAGTTCTTCATGCAC |
| Cg25c-3_fw | GGCTCCAGCGCTTCTAGTGGACTGGACTATCTCACCGGTATC |
| Cg25c-3_rv | CTTGCTGCCAGAGCCGGATCCCGAGGAGTTCTTCATGCAC |
| Vkg-1_fw | GATGACAAGCTAGCAAGTACGGCACCCAAGAGCCGCGG |
| Vkg-1_rv | ACCCGAGCTGGCAGAACTTCCGTTGCCTCGACGACGGCAAAC |
| Vkg-2_fw | GGAAGTTCTGCCAGCTCGGGTGCACCCAAGAGCCGCGG |
| Vkg-2_rv | TCCACTAGAAGCGCTGGAGCCGTTGCCTCGACGACGGCAAAC |
| Vkg-3_fw | GGCTCCAGCGCTTCTAGTGGAGCACCCAAGAGCCGCGG |
| Vkg-3_rv | CTTGCTGCCAGAGCCGGATCCGTTGCCTCGACGACGGCAAAC |
| mEmer_fw | GGATCCGGCTCTGGCAGCAAGGGCGAGGAGCTG |
| **For removal of mEmerald from single-chain NC1 trimers** | |
| pcDNAstop_fw | TAATAGATCGATAGGGCCCGTTTAAACC |
| mEm-lnk_rv | GCCAGAGCCGGATCCCGA |
