# Supplementary material for: Collagen IV of basement membranes: IV. Adaptive mechanism of collagen IV scaffold assembly in Drosophila
Source: J Biol Chem. 2023 Oct 27;299(12):105394. doi: 10.1016/j.jbc.2023.105394 (PMC10694668; doi:10.1016/j.jbc.2023.105394)
Supplement: Table S2 [file mmc3.docx]

Table S2. **Plasmids generated in this study.**

| **Plasmid name** | **Content** | **Type** |
| --- | --- | --- |
| pRcX_*Cg25cNC1* | NC1 of Cg25c | expression |
| pRcX_*VkgNC1* | NC1 of Vkg | expression |
| pcDNA_*CCC-mEmerald* | Single-chain polypeptide encoding NC1 sequences of Cg25c, Cg25c, and Cg25c and fluorescent protein mEmerald | cloning/expression |
| pcDNA_*VVV-mEmerald* | Single-chain polypeptide encoding NC1 sequences of Vkg, Vkg, and Vkg and fluorescent protein mEmerald | cloning/expression |
| pcDNA_*CVC-mEmerald* | Single-chain polypeptide encoding NC1 sequences of Cg25c, Vkg, and Cg25c and fluorescent protein mEmerald | cloning/expression |
| pcDNA_*VCV-mEmerald* | Single-chain polypeptide encoding NC1 sequences of Vkg, Cg25c, and Vkg and fluorescent protein mEmerald | cloning/expression |
| pValium10-roe_*UAS-CCC-mEemrald* | GAL4-UAS binary expression system for single-chain CCC fused to mEemrald | transgenic expression |
| pValium10-roe_*UAS-VVV-mEemrald* | GAL4-UAS binary expression system for single-chain VVV fused to mEemrald | transgenic expression |
| pValium10-roe_*UAS-CVC-mEemrald* | GAL4-UAS binary expression system for single-chain CVC fused to mEemrald | transgenic expression |
| pValium10-roe_*UAS-VCV-mEemrald* | GAL4-UAS binary expression system for single-chain VCV fused to mEemrald | transgenic expression |
| pcDNA-*CVC* | Single-chain polypeptide encoding NC1 sequences of Cg25c, Vkg, and Cg25c | expression |
| pcDNA-*VCV* | Single-chain polypeptide encoding NC1 sequences of Vkg, Cg25c, and Vkg | expression |
