# Supplementary material for: Collagen IV of basement membranes: IV. Adaptive mechanism of collagen IV scaffold assembly in Drosophila
Source: J Biol Chem. 2023 Oct 27;299(12):105394. doi: 10.1016/j.jbc.2023.105394 (PMC10694668; doi:10.1016/j.jbc.2023.105394)
Supplement: Table S3 [file mmc4.docx]

Table S3. **Data collection and refinement statistics**

| **Data collection statistics** | Recombinant (PDB ID 8TXN) | Native (PDB ID 8TYS) |
| --- | --- | --- |
| Wavelength (Å) | 0.97857 | 0.97857 |
| Resolution range (Å)^a^ | 52.05-1.75 (1.78-1.75) | 40.63-2.90 (3.08-2.90) |
| Space group | P6_3_ | P6_3_ |
| Unit cell dimensions (Å) | a=b=139.12, c=103.38 | a=b=145.21, c=106.49 |
| Unit cell angles (°) | α=β=90, γ=120 | α=β=90, γ=120 |
| Total reflections | 1,301,295 (62,110) | 326,147 (52,693) |
| Unique reflections | 114,327 (5,634) | 28,410 (4,562) |
| Redundancy | 11.4 (11.0) | 11.5 (11.6) |
| Completeness (%) | 100.0 (100.0) | 100.0 (100.0) |
| R_merge_ (%) | 8.4 (53.3) | 8.5 (45.0) |
| I/σI | 17.7 (4.3) | 17.3 (4.6) |
| **Refinement statistics** | | |
| Resolution range (Å)^a^ | 51.69-1.75 (1.79-1.75) | 40.63-2.90 (3.00-2.90) |
| Unique reflections used in refinement^b^ | 114,289 (7,960) | 28,388 (2,682) |
| R_work_ (%) | 14.2 (17.1) | 24.1 (34.0) |
| R_free_ (%)^c^ | 16.4 (20.9) | 27.7 (36.1) |
| Bond distance (Å)^d^ | 0.006 | 0.002 |
| Bond angle (°)^d^ | 0.889 | 0.521 |
| Chiral center (Å^3^)^d^ | 0.059 | 0.043 |
| Planar group (Å)^d^ | 0.007 | 0.004 |
| Dihedral angle (°)^d^ | 14.538 | 12.142 |
| Number of atoms (excluding hydrogens) | 11,574 | 10,472 |
| Protein atoms | 10,566 | 10,466 |
| Chloride ions | 4 | 4 |
| Divalent cations | 2 Mg^2+^ | 2 Ca^2+^ |
| Polyethylene glycol molecules/atoms | 15/135 | 0 |
| Water molecules | 429 | 0 |
| Mean B-value (Å^2^) | 23.37 | 78.82 |
| Mean protein B-value (Å^2^) | 22.59 | 78.83 |
| Mean divalent cation B-value (Å^2^) | 15.4 | 68.35 |
| Mean chloride ion B-value (Å^2^) | 18.64 | 66.69 |
| Mean polyethylene glycol molecule B-value (Å^2^) | 48.43 | - |
| Mean water B-value (Å^2^) | 28.73 | - |
| **Ramachandran statistics** | | |
| Favored (%) | 97.68 | 95.52 |
| Additionally allowed (%) | 2.17 | 4.33 |
| Outliers (%) | 0.15 | 0.15 |

^a^ Data for highest resolution shell are given in parentheses

^b^ Data cutoff σF=1.34

^c^ 1.75% (2,003 reflections) of recombinant and 4.70% (1,335 reflections) of native

^d^ Root mean square deviation
