# Supplementary material for: Collagen IV of basement membranes: IV. Adaptive mechanism of collagen IV scaffold assembly in Drosophila
Source: J Biol Chem. 2023 Oct 27;299(12):105394. doi: 10.1016/j.jbc.2023.105394 (PMC10694668; doi:10.1016/j.jbc.2023.105394)
Supplement: Table S4 [file mmc5.docx]

Table S4. **Coordination of chloride ions near the trimer-trimer interface.** Chloride ions are mainly coordinated by main-chain atoms of Cg25c chains and water molecules. Two ions are also coordinated by arginine residues from the Vkg chain of the opposite trimer (highlighted with a blue background). The other two ions cannot be coordinated similarly as Cg25c chains have Thr residues instead of Arg. Chain IDs: A – Cg25c (residues xxx of chain A in PDB 8TXN), B – Vkg (residues 1xxx of chain A in PDB 8TXN), C – Cg25c (residues 2xxx of chain A in PDB 8TXN), D – Cg25c (residues xxx of chain B in PDB 8TXN), E – Vkg (residues 1xxx of chain B in PDB 8TXN), F – Cg25c (residues 2xxx of chain B in PDB 8TXN). Chains A, B, and C are forming one trimer, chains D, E, and F are forming another trimer. The numbering of residues is given for the NC1 domain. To obtain a residue position in the full-length sequence add 1550 for Cg25c (chains A, C, D, and F) and 1510 for Vkg (chains B and E).

|  | Cl^-^ #1 |  |  | Cl^-^ #2 |  |  | Cl^-^ #3 |  |  | Cl^-^ #4 |
| --- | --- | --- | --- | --- | --- | --- | --- | --- | --- | --- |
| E:ARG 181[NH2] | 4.10 Å |  | B:ARG 181[NH2] | 4.20 Å |  | - |  |  | - |  |
| C:LEU 60[CD2] | 3.70 Å |  | F:LEU 60[C1] | 3.68 Å |  | A:LEU 60[CD2] | 3.78 Å |  | D:LEU 60[CD2] | 3.82 Å |
| C:ALA 74[CA] | 3.70 Å |  | F:ALA 74[CA] | 3.64 Å |  | A:ALA 74[CA] | 3.73 Å |  | D:ALA 74[CA] | 3.71 Å |
| C:ARG 76[N] | 3.27 Å |  | F:ARG 76[N] | 3.28 Å |  | A:ARG 76[N] | 3.17 Å |  | D:ARG 76[N] | 3.23 Å |
| C:ASP 78[N] | 3.43 Å |  | F:ASP 78[N] | 3.39 Å |  | A:ASP 78[N] | 3.32 Å |  | D:ASP 78[N] | 3.35 Å |
| H_2_O [O] | 3.11 Å |  | H_2_O [O] | 3.11 Å |  | H_2_O [O] | 3.01 Å |  | H_2_O [O] | 2.85 Å |
| H_2_O [O] | 3.11 Å |  | H_2_O [O] | 3.11 Å |  | H_2_O [O] | 3.06 Å |  | H_2_O [O] | 3.11 Å |
