# Supplementary material for: Collagen IV of basement membranes: IV. Adaptive mechanism of collagen IV scaffold assembly in Drosophila
Source: J Biol Chem. 2023 Oct 27;299(12):105394. doi: 10.1016/j.jbc.2023.105394 (PMC10694668; doi:10.1016/j.jbc.2023.105394)
Supplement: Table S5 [file mmc6.docx]

Table S5. **Magnesium ions environment at the trimer-trimer interface.** Residues coordinating magnesium ion or magnesium-clustered water molecules are highlighted with an orange background. Chain IDs: A – Cg25c (residues xxx of chain A in PDB 8TXN), B – Vkg (residues 1xxx of chain A in PDB 8TXN), C – Cg25c (residues 2xxx of chain A in PDB 8TXN), D – Cg25c (residues xxx of chain B in PDB 8TXN), E – Vkg (residues 1xxx of chain B in PDB 8TXN), F – Cg25c (residues 2xxx of chain B in PDB 8TXN). Chains A, B, and C are forming one trimer, chains D, E, and F are forming another trimer. The numbering of residues is given for the NC1 domain. To obtain a residue position in the full-length sequence add 1550 for Cg25c (chains A, C, D, and F) and 1510 for Vkg (chains B and E).

| Atom 1 | Distance, Å | Atom 2 | Distance to Mg^2+^, Å |  | Atom 1 | Distance, Å | Atom 2 | Distance to Mg^2+^, Å |
| --- | --- | --- | --- | --- | --- | --- | --- | --- |
|  |  | C:ASP 40[OD2] | 2.05 |  |  |  | F:ASP 40[OD2] | 2.15 |
| E:ASP 79[OD2] | 2.79 | H_2_O [O] | 2.09 |  | B:ASP 79[OD2] | 2.89 | H_2_O [O] | 2.11 |
| E:ASP 77[O] | 3.01 |  |  |  | B:ASP 77[O] | 2.89 |  |  |
| C:LYS 79[NZ] | 2.92 | H_2_O [O] | 2.13 |  | F:LYS 79[NZ] | 3.04 | H_2_O [O] | 2.13 |
| C:ASN 77[O] | 2.66 |  |  |  | F:ASN 77[O] | 2.72 |  |  |
| E:ASP 79[OD2] | 2.53 | H_2_O [O] | 2.11 |  | B:ASP 79[OD2] | 2.53 | H_2_O [O] | 2.17 |
| C:ASP 40[OD1] | 2.59 | H_2_O [O] | 2.15 |  | F:ASP 40[OD1] | 2.52 | H_2_O [O] | 2.04 |
| E:SER 40[OG] | 3.14 |  |  |  | B:SER 40[OG] | 3.33 |  |  |
| C:LYS 79[NZ] | 3.36 |  |  |  | F:LYS 79[NZ] | 3.38 |  |  |
|  |  | H_2_O [O] | 2.07 |  |  |  | H_2_O [O] | 2.04 |
