# Supplementary material for: Collagen IV of basement membranes: IV. Adaptive mechanism of collagen IV scaffold assembly in Drosophila
Source: J Biol Chem. 2023 Oct 27;299(12):105394. doi: 10.1016/j.jbc.2023.105394 (PMC10694668; doi:10.1016/j.jbc.2023.105394)
Supplement: Supplementary materials [file mmc7.docx]

Supporting Information to: **Collagen IV of basement membranes: IV. Adaptive mechanism of collagen IV scaffold assembly in *Drosophila*.**

***Authors***

Jacob A. Summers, Madison Yarbrough, Min Liu, W. Hayes McDonald, Billy G. Hudson, José C. Pastor-Pareja, Sergei P. Boudko

***Content***

Figure S1. 7S region in human and Drosophila collagen IV chains S-2

Figure S2. Tail sequences of Vkg chain S-3

Figure S3. Distance tree of NC1 domain sequences S-4

Figure S4. Sequence identities of Drosophila and human NC1 domains S-5

Figure S5. Identification of NC1 hexamer peak after collagenase digest and SEC S-6

Figure S6. The final purification step of NC1 hexamer from Drosophila pellet S-7

Figure S7. Chain identity of the purified NC1 hexamer of Drosophila S-8

Figure S8. Sequences of recombinantly produced NC1 monomers of *D. melanogaster* S-9

Figure S9. Size-exclusion chromatography of NC1 domains in chloride-free buffer S-10

Figure S10. Transient nature of Cg25c/Vkd trimers S-11

Figure S11. Sequence of single-chain CCC trimer fused with mEmerald S-12

Figure S12. Sequence of single-chain VVV trimer fused with mEmerald S-13

Figure S13. Sequence of single-chain CVC trimer fused with mEmerald S-14

Figure S14. Sequence of single-chain VCV trimer fused with mEmerald S-15

Figure S15. SEC of single-chain trimers fused with mEmerald S-16

Figure S16. Sequences of single-chain CVC and VCV trimers S-17

Figure S17. Affinity purification of single-chain CVC trimer S-18

Figure S18. Hexamer assembly in the presence of di- and tri-valent cations S-19

Figure S19. Typical gels for analysis of the hexamer assembly under various conditions S-20

Figure S20. Chloride effect on the hexamer assembly S-21

Figure S21. Drosophila NC1 domain organization is similar to mammalian S-22

Figure S22. Drosophila-unique residues for trimer association S-23

Figure S23. Clustering of Drosophila and human NC1 chains S-24

Figure S24. Polyethylene glycol (PEG) molecules in the crystal structure S-25

Figure S25. Drosophila NC1 hexamer surface topology and electrostatic potential S-26

Figure S26. Drosophila NC1 trimer surface topology and electrostatic potential S-27


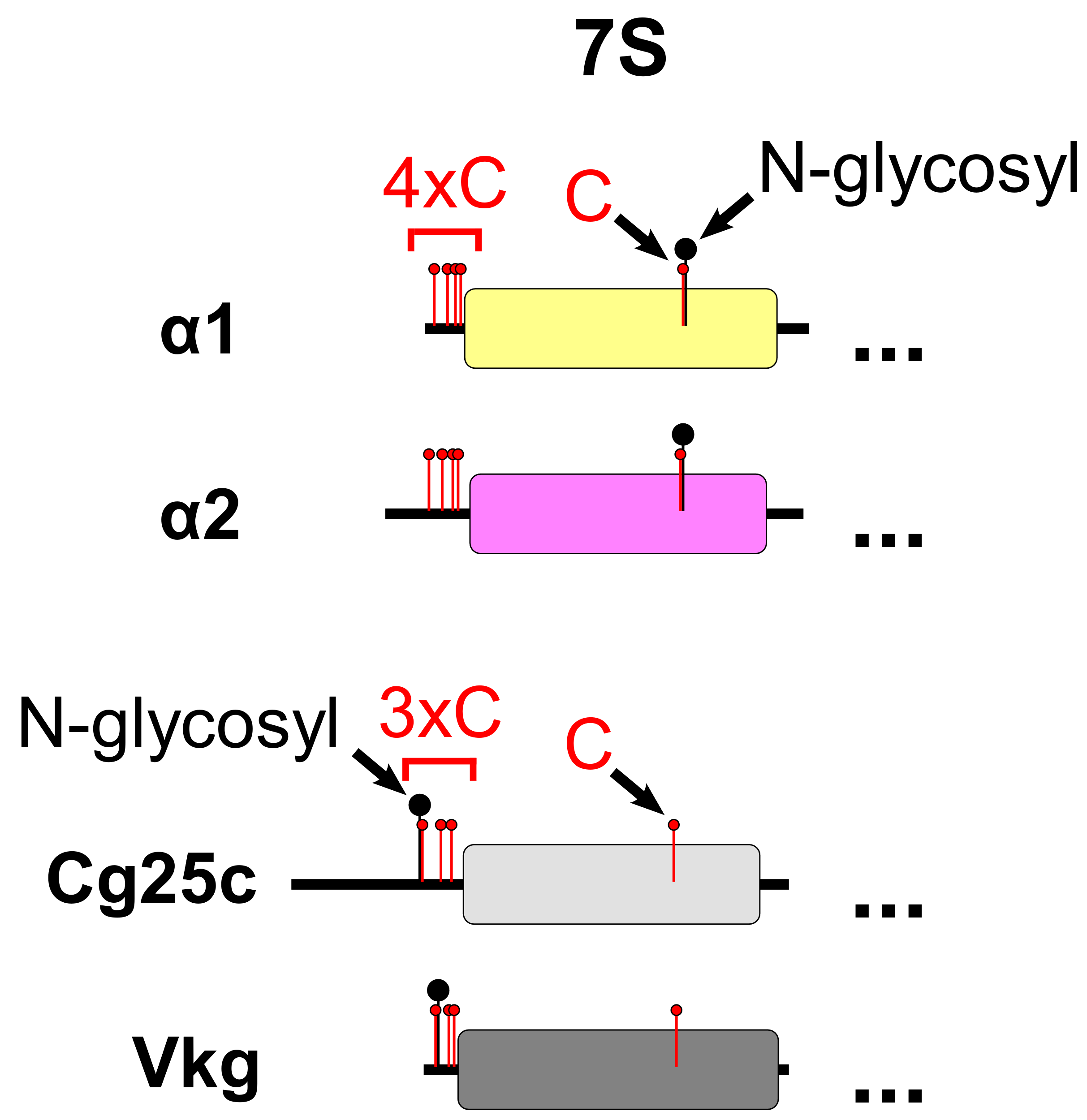


Figure S1. **7S region in human and *Drosophila* collagen IV chains.** Shown are 7S regions of human α1 and α2 chains and *Drosophila* Cg25c and Vkg chains. Bars represent triple-helical segments. Black lines represent non-triple-helical sequences. Cysteines are shown as red pins and potential sites for N-glycosylation of asparagine are shown as black pins.


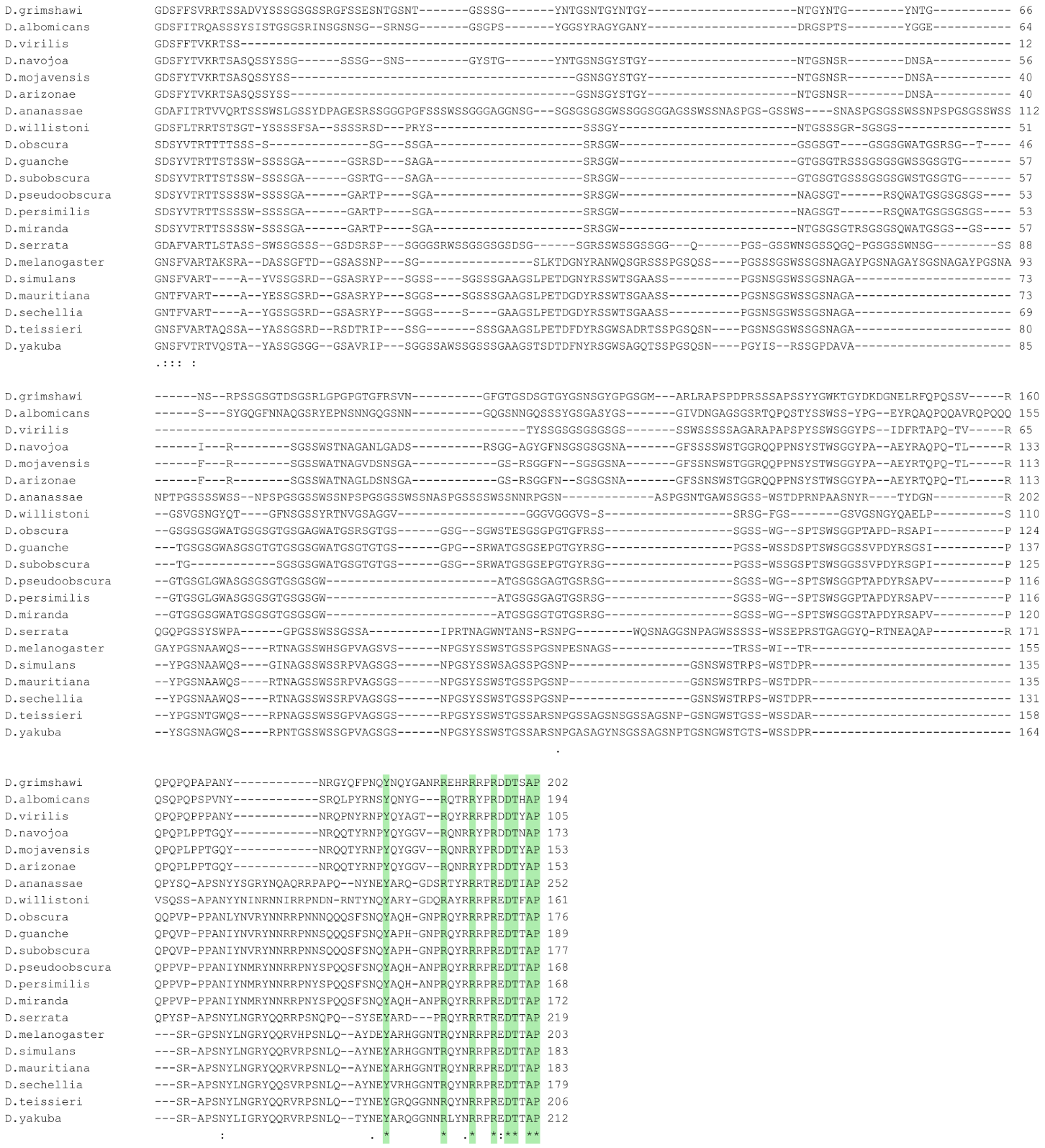


Figure S2. **Tail sequences of Vkg chain.** Multiple sequence alignment of the tails of Vkg genes from different Drosophila. Some conservation is only found at the very end of tail sequences (highlighted with a green background). Sequences derived from the NCBI protein database (https://www.ncbi.nlm.nih.gov/protein/) with the following IDs: XP_014761464.1 *D. ananassae*, XP_034128513.1 for *D. guanche*, XP_034669321.1 for *D. subobscura*, XP_022221956.2 for *D. obscura*, XP_001355956.3 for *D. pseudoobscura*, XP_017151781.2 for *D. miranda*, XP_026843831.1 for *D. persimilis*, XP_002067184.1 for *D. willistoni*, XP_020806048.1 for *D. serrata*, XP_033174114.1 for *D. mauritiana*, XP_039148274.1 for *D. simulans*, XP_032581231.1 for *D. sechellia*, XP_043648502.1 for *D. teissieri*, XP_002088686.2 for *D. yakuba*, XP_001989010.2 for *D. grimshawi*, XP_002051847.2 for *D. virilis*, XP_030243738.1 for *D. navojoa*, XP_002001673.1 for *D. mojavensis*, XP_017858025.1 for *D.  arizonae*.


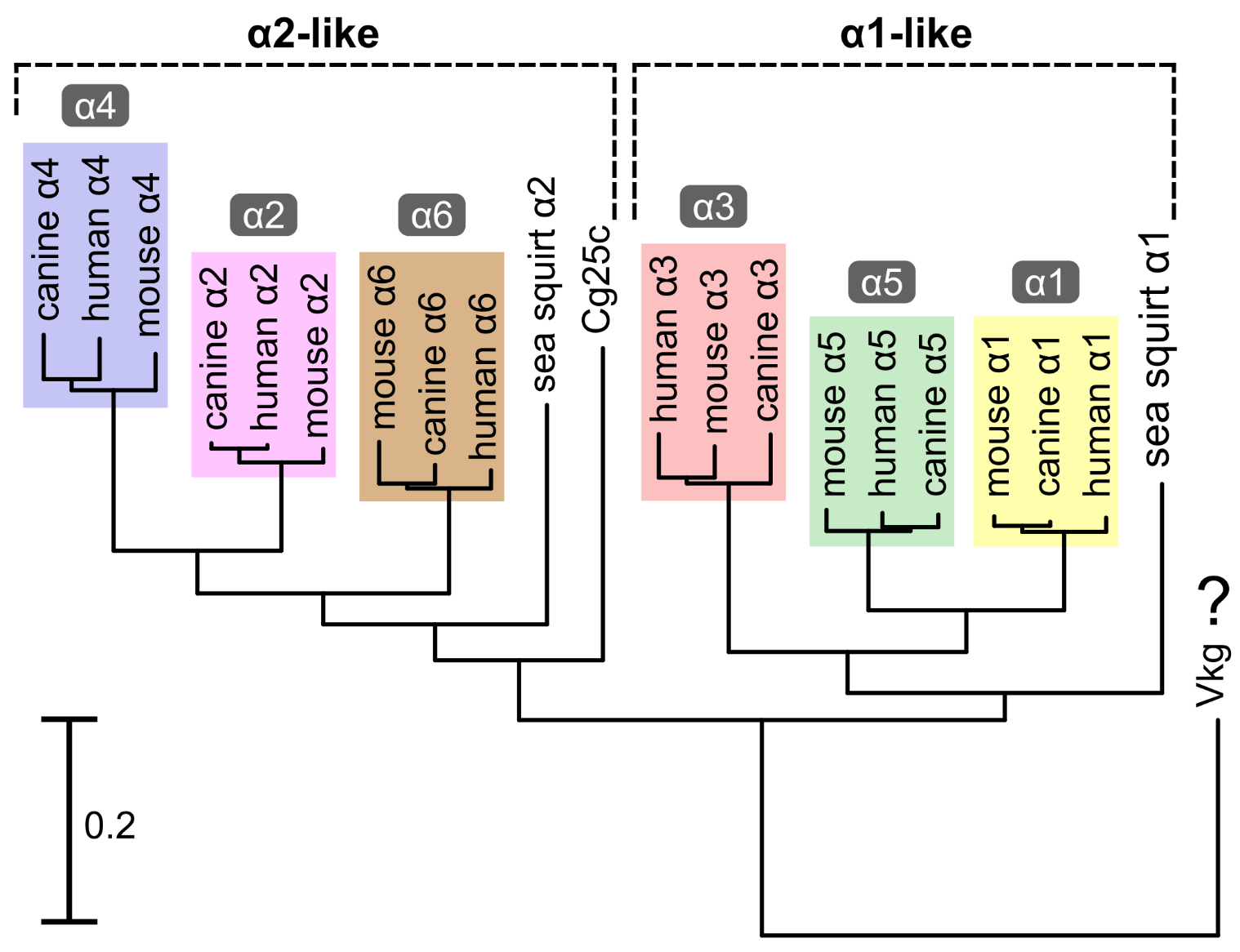


Figure S3. **Distance tree results of protein-protein BLAST multiple alignments of NC1 domain sequences.** Distance tree for human, mouse, canine, seq squirt, and drosophila sequences of the NC1 domain was generated using protein-protein BLAST multiple alignments. While Cg25c was assigned to an α2-like group, Vkg was not clustered to any group. NC1 sequences were derived from UniProt database: human α1 (P02462), α2 (P08572), α3 (Q01955), α4 (P53420), α5 (P29400), and α6 (Q14031); mouse α1 (P02463), α2 (P08122), α3 (Q9QZS0), α4 (Q9QZR9), α5 (Q63ZW6), and α6 (B1AVK5); canine α1 (A0A8I3S0U4), α2 (A0A8P0N8N5), α3 (A0A8I3NM06), α4 (A0A8I3NXU0), α5 (A0A8C0SD56), and α6 (A0A8P0NPC6); sea squirt α1 (F6VJG0) and α2 (F6WTL7); fruit fly Cg25c (P08120) and Vkg (Q9VMV5).


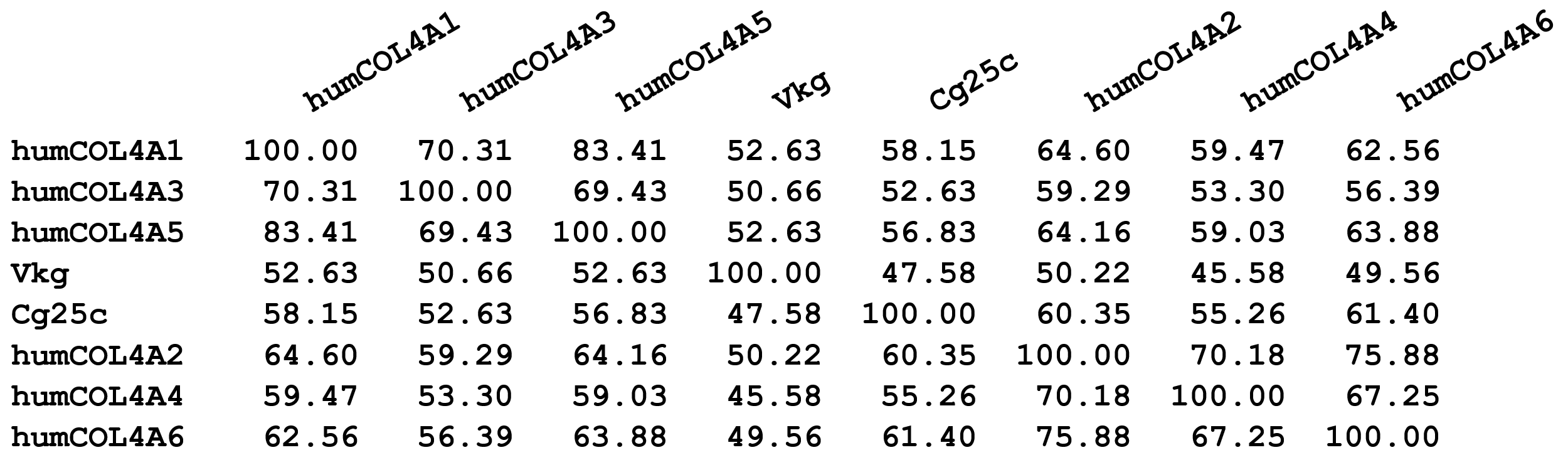


Figure S4. **Sequence identities of Drosophila and human NC1 domains.** The table shows identity scores for pairwise comparisons of chains. Vkg has slightly higher identity scores to α1-like chains, whereas Cg25c is slightly more identical to α2-like chains. Of note is that despite the higher identity of Cg25c to α2-like chains, it also shows a higher identity to α1-like chains than Vkg. The percent identity matrix was created by Clustal 2.1 (https://www.ebi.ac.uk/Tools/msa/clustalo/).


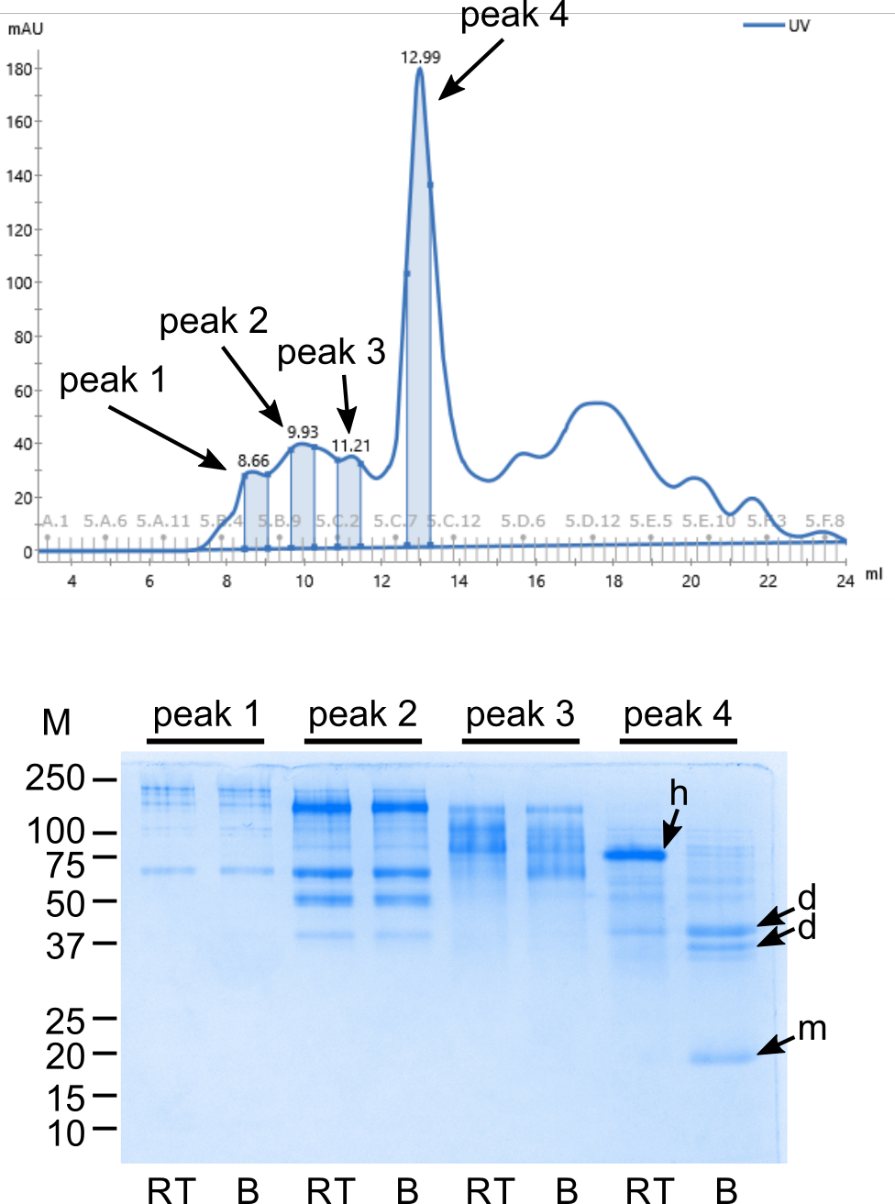


Figure S5. **Identification of NC1 hexamer peak after collagenase digest and SEC.** The SEC profile demonstrated a major peak at 12.99 ml (peak 4), whose position is similar to a mammalian NC1 hexamer. All preceding peaks (peaks 1-3), which correspond to higher apparent molecular size, were also analyzed to detect potential “tail”-containing isoforms of Vkg. None of these fractions seemed to contain detectable amounts of monomeric (m) or dimeric (d) forms of “tail’-free Cg25c chain that would accompany a “tailed” Vkg isoform, as well as no signs of SDS-resistant hexamer (h) for non-boiled samples. RT – room-temperature prepared SDS sample, B – boiled SDS sample.


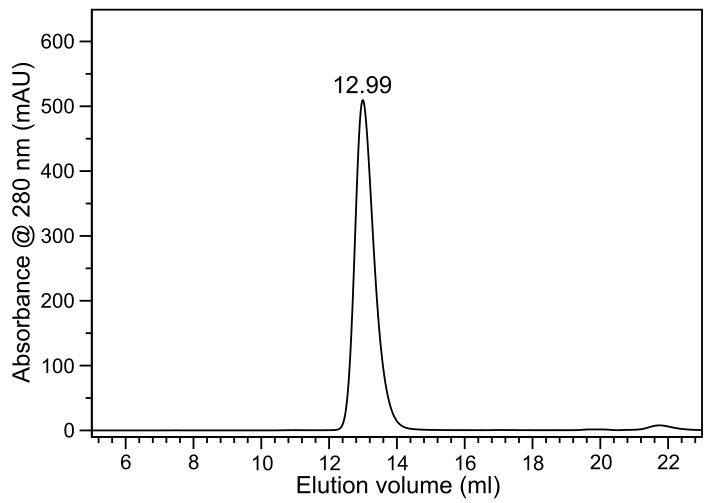


Figure S6. **The final purification step of NC1 hexamer from *Drosophila* pellet**. Size-exclusion chromatography after the hydroxyapatite (HAP) column. A single peak at 12.99 ml corresponds to the NC1 hexamer of *Drosophila*.


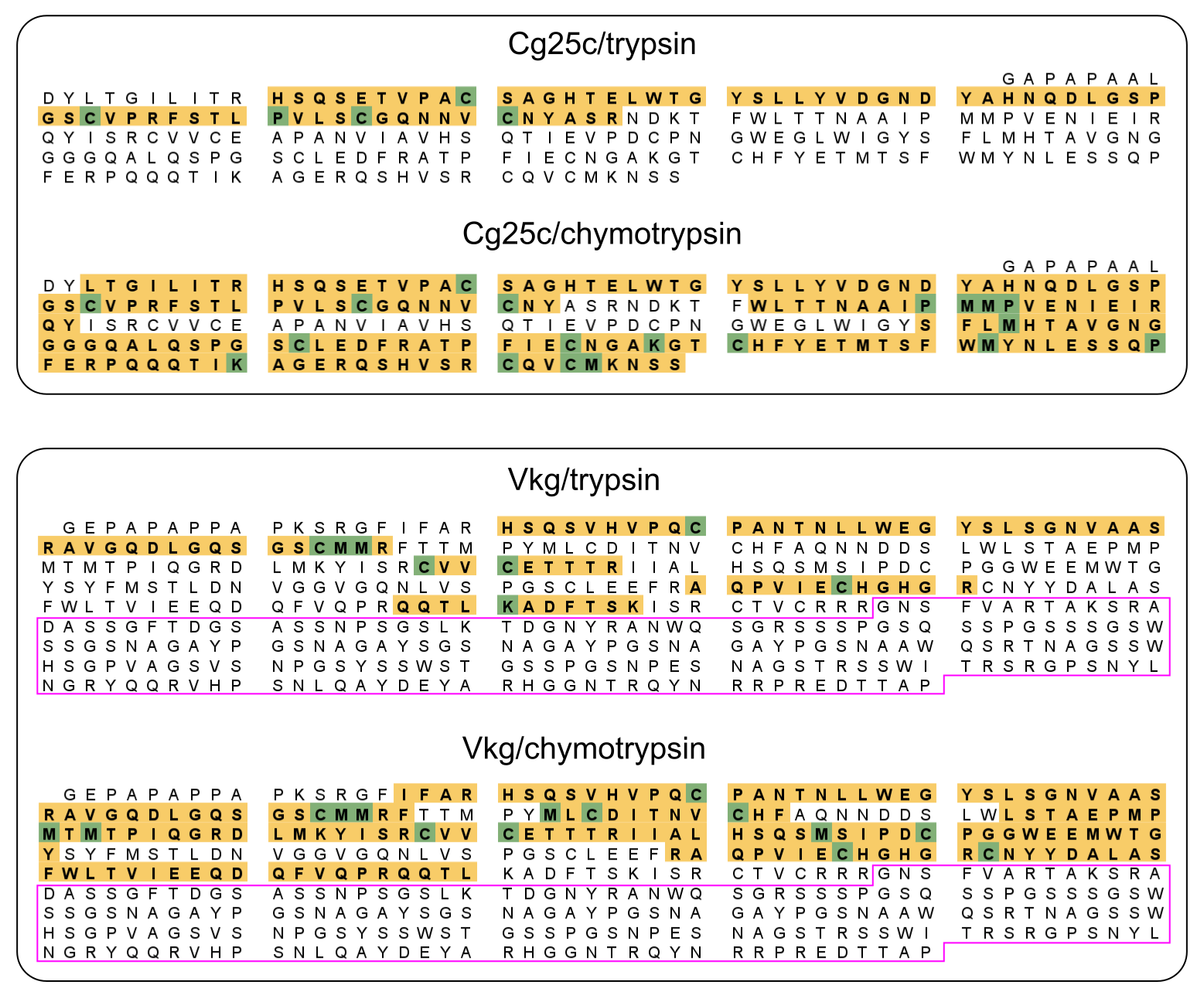


Figure S7. **Chain identity of the purified NC1 hexamer of Drosophila.** Peptides from Cg25c and Vkg were detected after in-gel trypsin and chymotrypsin digests followed by MS/MS analysis. The coverage of identified peptides is highlighted with a yellow background. Hydroxylation of lysine residues, carbamidomethylation of cysteine residues, and oxidation of methionine residues are highlighted with a green background. Visualized by Scaffold Viewer v5 (Proteome Software). The tail sequence of the Vkg chain had no coverage (magenta frame).

Flag-tagged Cg25c NC1:

MRAWIFFLLCLAGRALAAPLA**DYKDDDDK**LA**GEPGAPAPAALDYLTGILITRHSQSETVPACSAGHTELWTGYSLLYVDGNDYAHNQDLGSPGSCVPRFSTLPVLSCGQNNVCNYASRNDKTFWLTTNAAIPMMPVENIEIRQYISRCVVCEAPANVIAVHSQTIEVPDCPNGWEGLWIGYSFLMHTAVGNGGGGQALQSPGSCLEDFRATPFIECNGAKGTCHFYETMTSFWMYNLESSQPFERPQQQTIKAGERQSHVSRCQVCMKNSS***

His-tagged Cg25c NC1:

MRAWIFFLLCLAGRALAAPGS**HHHHHH**GSGLVPRGSGLA**GEPGAPAPAALDYLTGILITRHSQSETVPACSAGHTELWTGYSLLYVDGNDYAHNQDLGSPGSCVPRFSTLPVLSCGQNNVCNYASRNDKTFWLTTNAAIPMMPVENIEIRQYISRCVVCEAPANVIAVHSQTIEVPDCPNGWEGLWIGYSFLMHTAVGNGGGGQALQSPGSCLEDFRATPFIECNGAKGTCHFYETMTSFWMYNLESSQPFERPQQQTIKAGERQSHVSRCQVCMKNSS***

Flag-tagged Vkg NC1:

MRAWIFFLLCLAGRALAAPLA**DYKDDDDK**LA**GEPAPAPPAPKSRGFIFARHSQSVHVPQCPANTNLLWEGYSLSGNVAASRAVGQDLGQSGSCMMRFTTMPYMLCDITNVCHFAQNNDDSLWLSTAEPMPMTMTPIQGRDLMKYISRCVVCETTTRIIALHSQSMSIPDCPGGWEEMWTGYSYFMSTLDNVGGVGQNLVSPGSCLEEFRAQPVIECHGHGRCNYYDALASFWLTVIEEQDQFVQPRQQTLKADFTSKISRCTVCRRRGNS***

His-tagged Vkg NC1:

MRAWIFFLLCLAGRALAAPGS**HHHHHH**GSGLVPRGSGLA**GEPAPAPPAPKSRGFIFARHSQSVHVPQCPANTNLLWEGYSLSGNVAASRAVGQDLGQSGSCMMRFTTMPYMLCDITNVCHFAQNNDDSLWLSTAEPMPMTMTPIQGRDLMKYISRCVVCETTTRIIALHSQSMSIPDCPGGWEEMWTGYSYFMSTLDNVGGVGQNLVSPGSCLEEFRAQPVIECHGHGRCNYYDALASFWLTVIEEQDQFVQPRQQTLKADFTSKISRCTVCRRRGNS***

Figure S8. **Sequences of recombinantly produced NC1 monomers of *D. melanogaster*.** The underlined sequence is a SPARC signal peptide. Bold blue – **Flag-tag**. Bold green – **His-tag**. Bold black – **NC1 sequences** of *Cg25c* (residues 1540-1779, UniProt entry P08120) and *Viking* (residues 1502-1740, UniProt entry Q9VMV5).


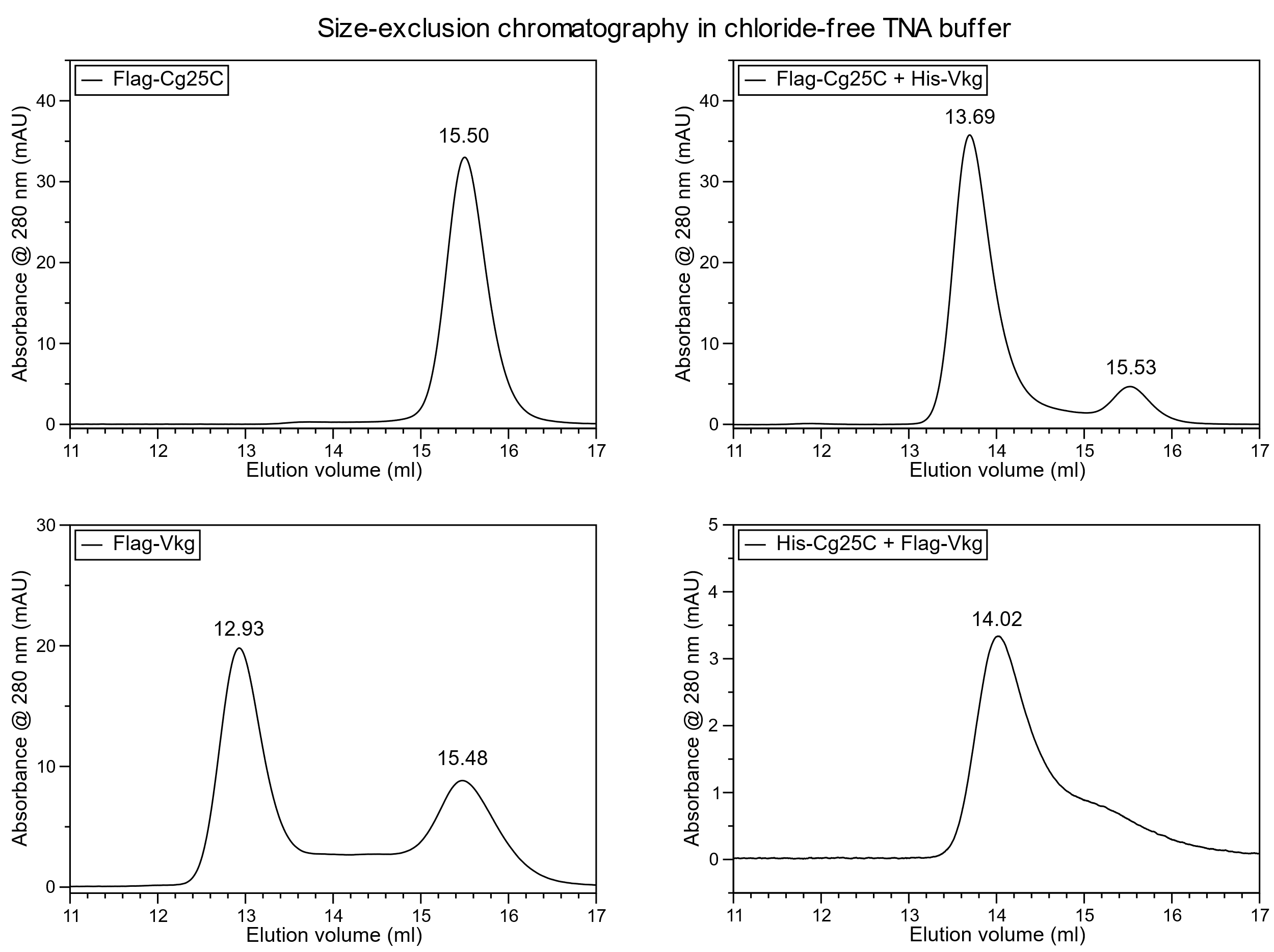


Figure S9. **Size-exclusion chromatography of NC1 domains in chloride-free buffer.** Fractions corresponding to the highest oligomeric state after the size-exclusion chromatography in the presence of chloride (TBS buffer) were pooled, concentrated, and re-run on a size-exclusion column equilibrated with TNA buffer. Cg25c “monomer” remained a single monomer peak, whereas Vkg “tetramer” peak revealed “tetramer” and presumably “monomer” peaks. “Trimers” of co-expressed Cg25c and Vkg chains remained “trimers” with the appearance of a subfraction corresponding to a “monomer”.


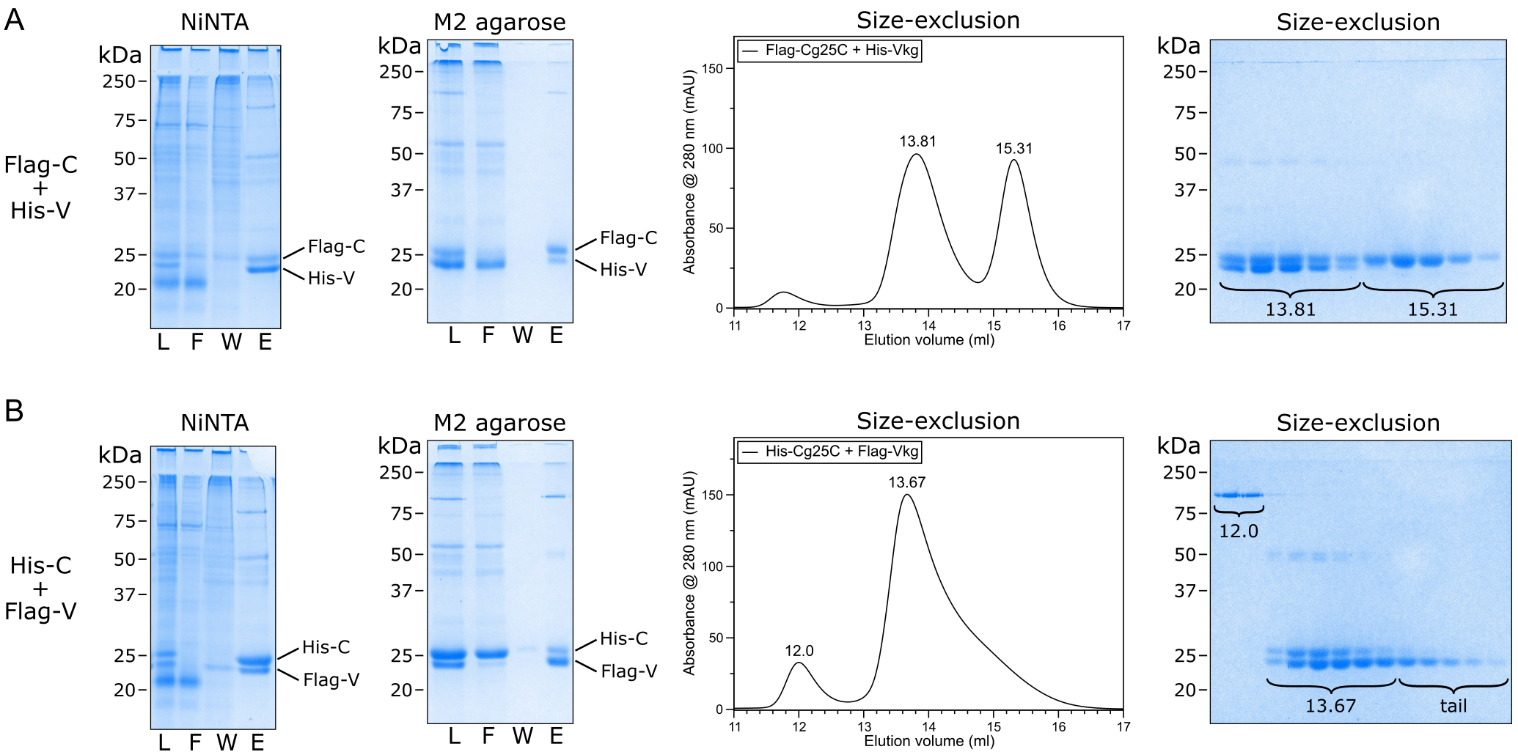


Figure S10. **Transient nature of Cg25c/Vkd trimers.** Two combinations of Cg25c and Vkg NC1 domains were co-expressed in expiCHO-S cells and serially purified over Ni-NTA column (His-tag affinity), M2 agarose (anti-Flag), and finally a size-exclusion chromatography of the elution fractions. The affinity purification fractions are load (L), flow-through (F), wash (W), and elution (E). (**A**) The first combination was Flag-tagged Cg25c and His-tagged Vkg NC1. (**B**) The second was His-tagged Cg25c and Flag-tagged Vkg. Note that second affinity purifications led to leakage of the His-tagged chain in the flow-through fraction, which could be a dissociation from the heterotrimer or an unbound homotrimer. In any case, if Cg25c and Vkg NC1 domains would form a stable complex, then two rounds of affinity purification using chain-specific tags would result in the removal of any non-assembled and homo-oligomeric complexes, but not the stable heterotrimer. Nevertheless, the size-exclusion chromatography reveals either a separate “monomer” peak in the case of Flag-tagged Cg25c and His-tagged Vkg NC1 complex or an asymmetric tail of His-tagged Cg25c and Flag-tagged Vkg. A peak around 12ml contained an impurity as revealed on a gel. Gel analysis confirms the hetero-complex nature of the peaks at around 13.67-13.81 and the monomeric nature of the peak at 15.3 ml or asymmetric shoulder. For both experiments, an apparent composition of the trimer peak is ~1:2 ratio of Cg25c:Vkg.

CCC-mEmerald:

MRAWIFFLLCLAGRALAAPLA**DYKDDDDK**LAST**LDYLTGILITRHSQSETVPACSAGHTELWTGYSLLYVDGNDYAHNQDLGSPGSCVPRFSTLPVLSCGQNNVCNYASRNDKTFWLTTNAAIPMMPVENIEIRQYISRCVVCEAPANVIAVHSQTIEVPDCPNGWEGLWIGYSFLMHTAVGNGGGGQALQSPGSCLEDFRATPFIECNGAKGTCHFYETMTSFWMYNLESSQPFERPQQQTIKAGERQSHVSRCQVCMKNSS**

GSSASSG

**LDYLTGILITRHSQSETVPACSAGHTELWTGYSLLYVDGNDYAHNQDLGSPGSCVPRFSTLPVLSCGQNNVCNYASRNDKTFWLTTNAAIPMMPVENIEIRQYISRCVVCEAPANVIAVHSQTIEVPDCPNGWEGLWIGYSFLMHTAVGNGGGGQALQSPGSCLEDFRATPFIECNGAKGTCHFYETMTSFWMYNLESSQPFERPQQQTIKAGERQSHVSRCQVCMKNSS**

GSSASSG

**LDYLTGILITRHSQSETVPACSAGHTELWTGYSLLYVDGNDYAHNQDLGSPGSCVPRFSTLPVLSCGQNNVCNYASRNDKTFWLTTNAAIPMMPVENIEIRQYISRCVVCEAPANVIAVHSQTIEVPDCPNGWEGLWIGYSFLMHTAVGNGGGGQALQSPGSCLEDFRATPFIECNGAKGTCHFYETMTSFWMYNLESSQPFERPQQQTIKAGERQSHVSRCQVCMKNSS**

GSGSGS

**KGEELFTGVVPILVELDGDVNGHKFSVSGEGEGDATYGKLTLKFICTTGKLPVPWPTLVTTLTYGVQCFARYPDHMKQHDFFKSAMPEGYVQERTIFFKDDGNYKTRAEVKFEGDTLVNRIELKGIDFKEDGNILGHKLEYNYNSHKVYITADKQKNGIKVNFKTRHNIEDGSVQLADHYQQNTPIGDGPVLLPDNHYLSTQSKLSKDPNEKRDHMVLLEFVTAAGITLGMDELYK***

Figure S11. **The sequence of single-chain CCC trimer fused with mEmerald.** The underlined sequence is a SPARC signal peptide. Bold blue – **Flag-tag**. Bold orange – NC1 sequence of ***Cg25c*** (residues 1550-1779, UniProt entry P08120). Bold green – **mEmerald**.

VVV-mEmerald:

MRAWIFFLLCLAGRALAAPLA**DYKDDDDK**LAST**APKSRGFIFARHSQSVHVPQCPANTNLLWEGYSLSGNVAASRAVGQDLGQSGSCMMRFTTMPYMLCDITNVCHFAQNNDDSLWLSTAEPMPMTMTPIQGRDLMKYISRCVVCETTTRIIALHSQSMSIPDCPGGWEEMWTGYSYFMSTLDNVGGVGQNLVSPGSCLEEFRAQPVIECHGHGRCNYYDALASFWLTVIEEQDQFVQPRQQTLKADFTSKISRCTVCRRRGN**

GSSASSG

**APKSRGFIFARHSQSVHVPQCPANTNLLWEGYSLSGNVAASRAVGQDLGQSGSCMMRFTTMPYMLCDITNVCHFAQNNDDSLWLSTAEPMPMTMTPIQGRDLMKYISRCVVCETTTRIIALHSQSMSIPDCPGGWEEMWTGYSYFMSTLDNVGGVGQNLVSPGSCLEEFRAQPVIECHGHGRCNYYDALASFWLTVIEEQDQFVQPRQQTLKADFTSKISRCTVCRRRGN**

GSSASSG

**APKSRGFIFARHSQSVHVPQCPANTNLLWEGYSLSGNVAASRAVGQDLGQSGSCMMRFTTMPYMLCDITNVCHFAQNNDDSLWLSTAEPMPMTMTPIQGRDLMKYISRCVVCETTTRIIALHSQSMSIPDCPGGWEEMWTGYSYFMSTLDNVGGVGQNLVSPGSCLEEFRAQPVIECHGHGRCNYYDALASFWLTVIEEQDQFVQPRQQTLKADFTSKISRCTVCRRRGN**

GSGSGS

**KGEELFTGVVPILVELDGDVNGHKFSVSGEGEGDATYGKLTLKFICTTGKLPVPWPTLVTTLTYGVQCFARYPDHMKQHDFFKSAMPEGYVQERTIFFKDDGNYKTRAEVKFEGDTLVNRIELKGIDFKEDGNILGHKLEYNYNSHKVYITADKQKNGIKVNFKTRHNIEDGSVQLADHYQQNTPIGDGPVLLPDNHYLSTQSKLSKDPNEKRDHMVLLEFVTAAGITLGMDELYK***

Figure S12. **The sequence of single-chain VVV trimer fused with mEmerald.** The underlined sequence is a SPARC signal peptide. Bold blue – **Flag-tag**. Bold purple - NC1 sequence of ***Viking*** (residues 1510-1739, UniProt entry Q9VMV5). Bold green – **mEmerald**.

CVC-mEmerald:

MRAWIFFLLCLAGRALAAPLA**DYKDDDDK**LAST**LDYLTGILITRHSQSETVPACSAGHTELWTGYSLLYVDGNDYAHNQDLGSPGSCVPRFSTLPVLSCGQNNVCNYASRNDKTFWLTTNAAIPMMPVENIEIRQYISRCVVCEAPANVIAVHSQTIEVPDCPNGWEGLWIGYSFLMHTAVGNGGGGQALQSPGSCLEDFRATPFIECNGAKGTCHFYETMTSFWMYNLESSQPFERPQQQTIKAGERQSHVSRCQVCMKNSS**

GSSASSG

**APKSRGFIFARHSQSVHVPQCPANTNLLWEGYSLSGNVAASRAVGQDLGQSGSCMMRFTTMPYMLCDITNVCHFAQNNDDSLWLSTAEPMPMTMTPIQGRDLMKYISRCVVCETTTRIIALHSQSMSIPDCPGGWEEMWTGYSYFMSTLDNVGGVGQNLVSPGSCLEEFRAQPVIECHGHGRCNYYDALASFWLTVIEEQDQFVQPRQQTLKADFTSKISRCTVCRRRGN**

GSSASSG

**LDYLTGILITRHSQSETVPACSAGHTELWTGYSLLYVDGNDYAHNQDLGSPGSCVPRFSTLPVLSCGQNNVCNYASRNDKTFWLTTNAAIPMMPVENIEIRQYISRCVVCEAPANVIAVHSQTIEVPDCPNGWEGLWIGYSFLMHTAVGNGGGGQALQSPGSCLEDFRATPFIECNGAKGTCHFYETMTSFWMYNLESSQPFERPQQQTIKAGERQSHVSRCQVCMKNSS**

GSGSGS

**KGEELFTGVVPILVELDGDVNGHKFSVSGEGEGDATYGKLTLKFICTTGKLPVPWPTLVTTLTYGVQCFARYPDHMKQHDFFKSAMPEGYVQERTIFFKDDGNYKTRAEVKFEGDTLVNRIELKGIDFKEDGNILGHKLEYNYNSHKVYITADKQKNGIKVNFKTRHNIEDGSVQLADHYQQNTPIGDGPVLLPDNHYLSTQSKLSKDPNEKRDHMVLLEFVTAAGITLGMDELYK***

Figure S13. **The sequence of single-chain CVC trimer fused with mEmerald.** The underlined sequence is a SPARC signal peptide. Bold blue – **Flag-tag**. Bold orange – NC1 sequence of ***Cg25c*** (residues 1550-1779, UniProt entry P08120) and bold purple – ***Viking*** (residues 1510-1739, UniProt entry Q9VMV5). Bold green – **mEmerald**.

VCV-mEmerald:

MRAWIFFLLCLAGRALAAPLA**DYKDDDDK**LAST**APKSRGFIFARHSQSVHVPQCPANTNLLWEGYSLSGNVAASRAVGQDLGQSGSCMMRFTTMPYMLCDITNVCHFAQNNDDSLWLSTAEPMPMTMTPIQGRDLMKYISRCVVCETTTRIIALHSQSMSIPDCPGGWEEMWTGYSYFMSTLDNVGGVGQNLVSPGSCLEEFRAQPVIECHGHGRCNYYDALASFWLTVIEEQDQFVQPRQQTLKADFTSKISRCTVCRRRGN**

GSSASSG

**LDYLTGILITRHSQSETVPACSAGHTELWTGYSLLYVDGNDYAHNQDLGSPGSCVPRFSTLPVLSCGQNNVCNYASRNDKTFWLTTNAAIPMMPVENIEIRQYISRCVVCEAPANVIAVHSQTIEVPDCPNGWEGLWIGYSFLMHTAVGNGGGGQALQSPGSCLEDFRATPFIECNGAKGTCHFYETMTSFWMYNLESSQPFERPQQQTIKAGERQSHVSRCQVCMKNSS**

GSSASSG

**APKSRGFIFARHSQSVHVPQCPANTNLLWEGYSLSGNVAASRAVGQDLGQSGSCMMRFTTMPYMLCDITNVCHFAQNNDDSLWLSTAEPMPMTMTPIQGRDLMKYISRCVVCETTTRIIALHSQSMSIPDCPGGWEEMWTGYSYFMSTLDNVGGVGQNLVSPGSCLEEFRAQPVIECHGHGRCNYYDALASFWLTVIEEQDQFVQPRQQTLKADFTSKISRCTVCRRRGN**

GSGSGS

**KGEELFTGVVPILVELDGDVNGHKFSVSGEGEGDATYGKLTLKFICTTGKLPVPWPTLVTTLTYGVQCFARYPDHMKQHDFFKSAMPEGYVQERTIFFKDDGNYKTRAEVKFEGDTLVNRIELKGIDFKEDGNILGHKLEYNYNSHKVYITADKQKNGIKVNFKTRHNIEDGSVQLADHYQQNTPIGDGPVLLPDNHYLSTQSKLSKDPNEKRDHMVLLEFVTAAGITLGMDELYK***

Figure S14. **The sequence of single-chain VCV trimer fused with mEmerald.** The underlined sequence is a SPARC signal peptide. Bold blue – **Flag-tag**. Bold purple - NC1 sequence of ***Viking*** (residues 1510-1739, UniProt entry Q9VMV5) and bold orange – NC1 sequence of ***Cg25c*** (residues 1550-1779, UniProt entry P08120). Bold green – **mEmerald**.


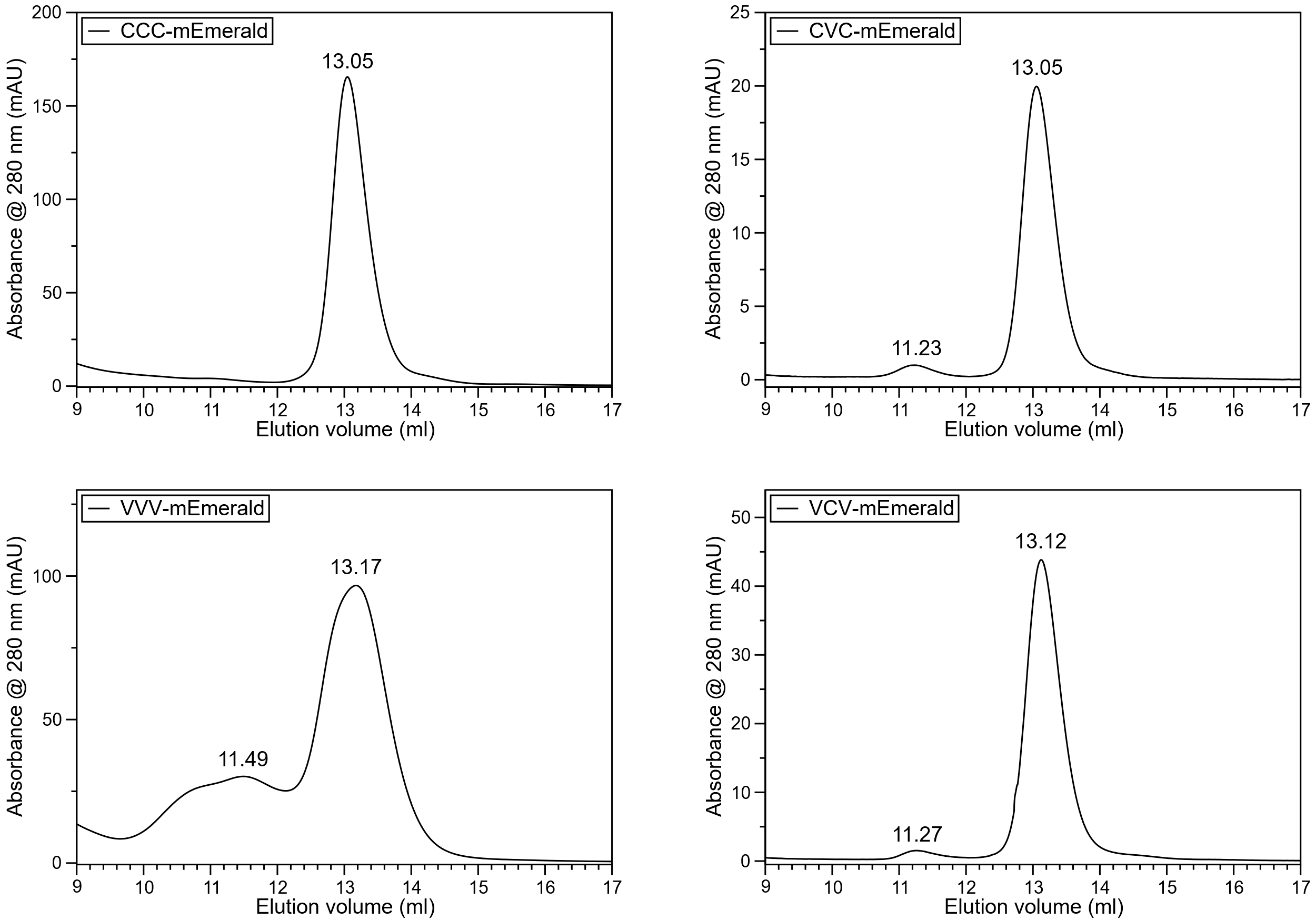


Figure S15. **SEC of single-chain NC1 trimers fused with mEmerald.** Four constructs, CCC, VVV, CVC, and VCV representing all possible variants of NC1 trimer, were expressed as fusions with Flag-tag and fluorescent protein mEmerald in expiCHO-S cells and purified on M2-agarose (anti-Flag) in the presence of chloride (TBS buffer). The affinity-purified material was run over the size-exclusion chromatography. All four constructs revealed a major peak at ~13 ml, which corresponds to a non-associated single-chain trimer with mEmerald fusion. VVV construct also revealed a tendency to promiscuous oligomerization by the appearance of additional peaks and broadening of the major peak. CVC and VCV also revealed a small peak at ~ 11ml that potentially indicated the formation of a dimer (which would correspond to a native NC1 hexamer with two additional mEmerald moieties), but no further analysis was performed due to an insufficient amount of material.

CVC:

MRAWIFFLLCLAGRALAAPLA**DYKDDDDK**LAST**LDYLTGILITRHSQSETVPACSAGHTELWTGYSLLYVDGNDYAHNQDLGSPGSCVPRFSTLPVLSCGQNNVCNYASRNDKTFWLTTNAAIPMMPVENIEIRQYISRCVVCEAPANVIAVHSQTIEVPDCPNGWEGLWIGYSFLMHTAVGNGGGGQALQSPGSCLEDFRATPFIECNGAKGTCHFYETMTSFWMYNLESSQPFERPQQQTIKAGERQSHVSRCQVCMKNSS**

GSSASSG

**APKSRGFIFARHSQSVHVPQCPANTNLLWEGYSLSGNVAASRAVGQDLGQSGSCMMRFTTMPYMLCDITNVCHFAQNNDDSLWLSTAEPMPMTMTPIQGRDLMKYISRCVVCETTTRIIALHSQSMSIPDCPGGWEEMWTGYSYFMSTLDNVGGVGQNLVSPGSCLEEFRAQPVIECHGHGRCNYYDALASFWLTVIEEQDQFVQPRQQTLKADFTSKISRCTVCRRRGN**

GSSASSG

**LDYLTGILITRHSQSETVPACSAGHTELWTGYSLLYVDGNDYAHNQDLGSPGSCVPRFSTLPVLSCGQNNVCNYASRNDKTFWLTTNAAIPMMPVENIEIRQYISRCVVCEAPANVIAVHSQTIEVPDCPNGWEGLWIGYSFLMHTAVGNGGGGQALQSPGSCLEDFRATPFIECNGAKGTCHFYETMTSFWMYNLESSQPFERPQQQTIKAGERQSHVSRCQVCMKNSS**GSGSGS

VCV:

MRAWIFFLLCLAGRALAAPLA**DYKDDDDK**LAST**APKSRGFIFARHSQSVHVPQCPANTNLLWEGYSLSGNVAASRAVGQDLGQSGSCMMRFTTMPYMLCDITNVCHFAQNNDDSLWLSTAEPMPMTMTPIQGRDLMKYISRCVVCETTTRIIALHSQSMSIPDCPGGWEEMWTGYSYFMSTLDNVGGVGQNLVSPGSCLEEFRAQPVIECHGHGRCNYYDALASFWLTVIEEQDQFVQPRQQTLKADFTSKISRCTVCRRRGN**

GSSASSG

**LDYLTGILITRHSQSETVPACSAGHTELWTGYSLLYVDGNDYAHNQDLGSPGSCVPRFSTLPVLSCGQNNVCNYASRNDKTFWLTTNAAIPMMPVENIEIRQYISRCVVCEAPANVIAVHSQTIEVPDCPNGWEGLWIGYSFLMHTAVGNGGGGQALQSPGSCLEDFRATPFIECNGAKGTCHFYETMTSFWMYNLESSQPFERPQQQTIKAGERQSHVSRCQVCMKNSS**

GSSASSG

**APKSRGFIFARHSQSVHVPQCPANTNLLWEGYSLSGNVAASRAVGQDLGQSGSCMMRFTTMPYMLCDITNVCHFAQNNDDSLWLSTAEPMPMTMTPIQGRDLMKYISRCVVCETTTRIIALHSQSMSIPDCPGGWEEMWTGYSYFMSTLDNVGGVGQNLVSPGSCLEEFRAQPVIECHGHGRCNYYDALASFWLTVIEEQDQFVQPRQQTLKADFTSKISRCTVCRRRGN**GSGSG

Figure S16. **Sequences of single-chain CVC and VCV trimers.** The underlined sequence is a SPARC signal peptide. Bold blue – **Flag-tag**. Bold orange – NC1 sequence of ***Cg25c*** (residues 1550-1779, UniProt entry P08120) and bold purple - ***Viking*** (residues 1510-1739, UniProt entry Q9VMV5).


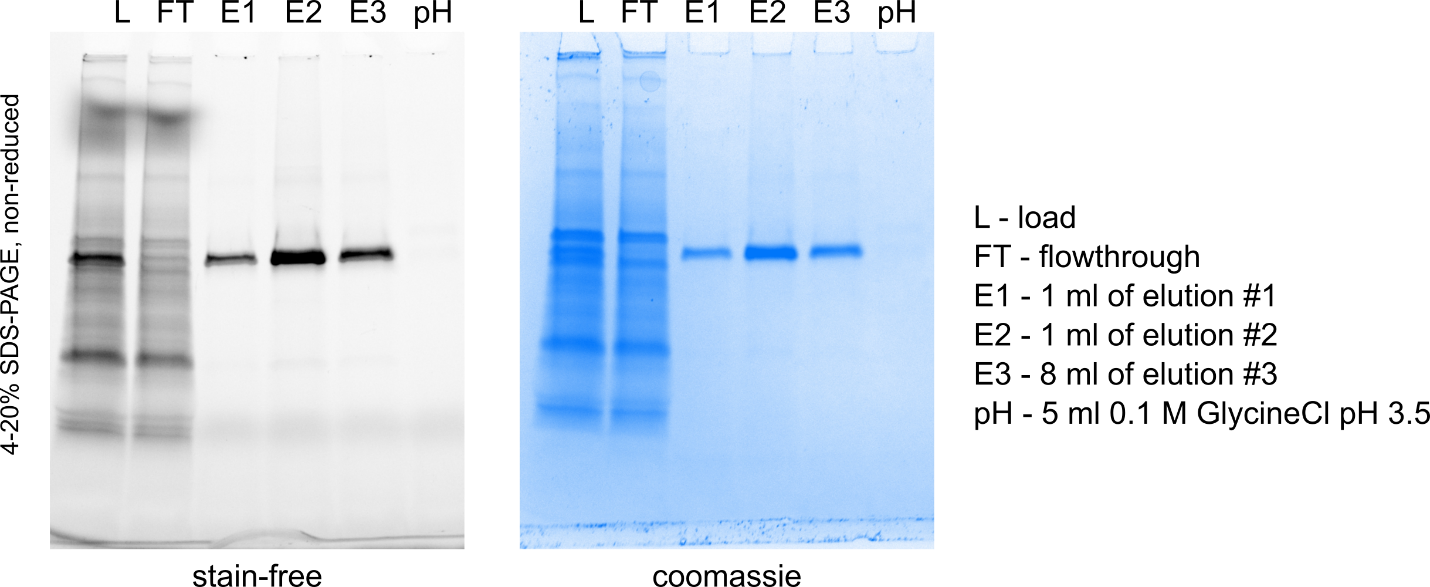


Figure S17. **Affinity purification of single-chain CVC trimer.** ExpiCHO cell suspension after transient expression of Flag-tagged CVC trimer was collected and centrifuged at 4,000 g for 10 minutes to collect medium with the secreted protein. The medium was dialyzed twice against 1 L of TBS and purified using 2 ml of M2 agarose column (MilliporeSigma). Elution was performed with 0.1 mg/ml Flag-peptide (MilliporeSigma). L – load; FT – flow through; E1-E3 – elution fractions with Flag-peptide. pH – elution with a low pH of 3.5.


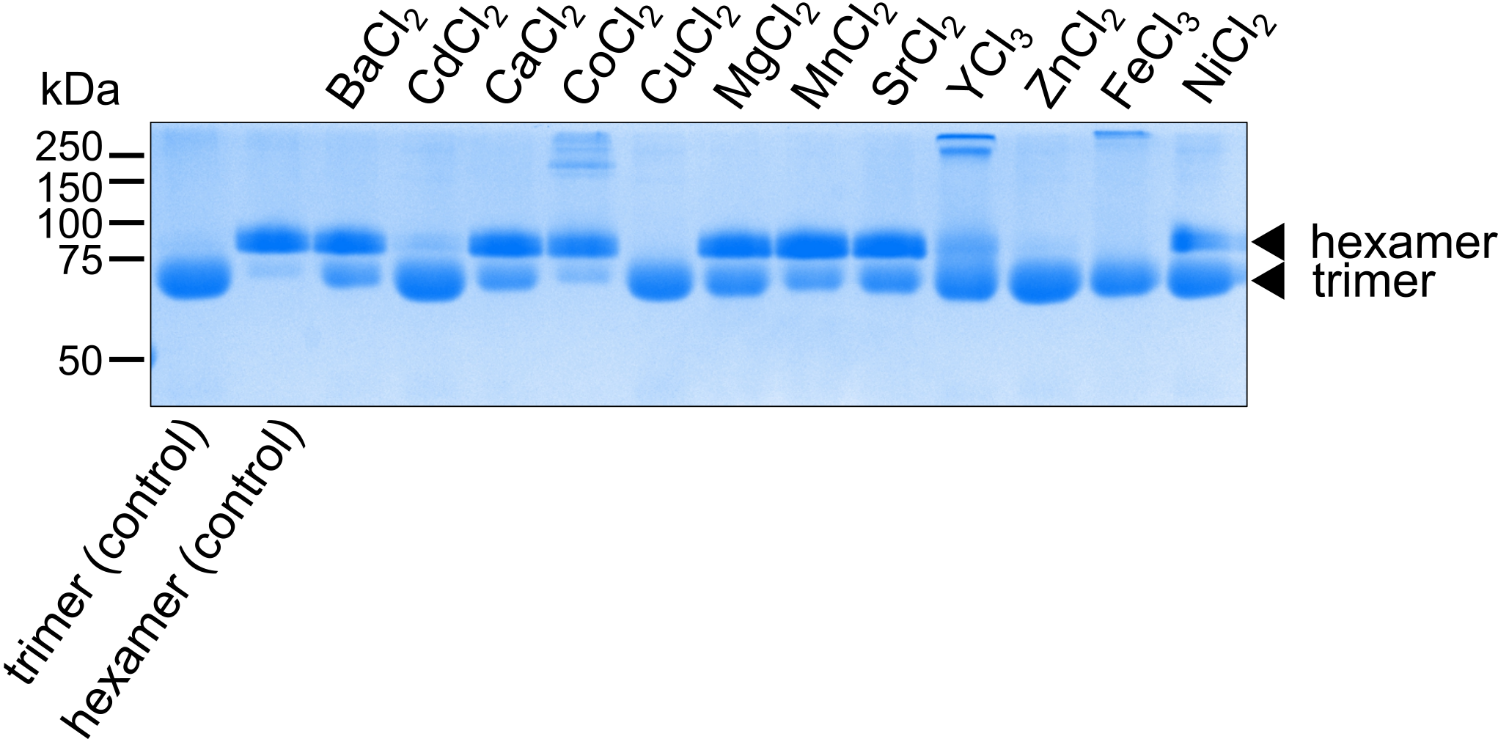


Figure S18. **Hexamer assembly in the presence of di- and trivalent cations.** Recombinant CVC trimer at 10 mg/ml concentration in 20 mM HEPES pH 7.5 was mixed with an equal volume of various cations at 10 mM concentration dissolved in the same buffer and incubated for 5 days at room temperature before analysis. The hexamer assembly was detected as the appearance of an SDS-resistant band. The samples were analyzed on 12% SDS-PAGE (coomassie stain). The cations were from the Additive Screen (Hampton Research, stock solutions A1-12).


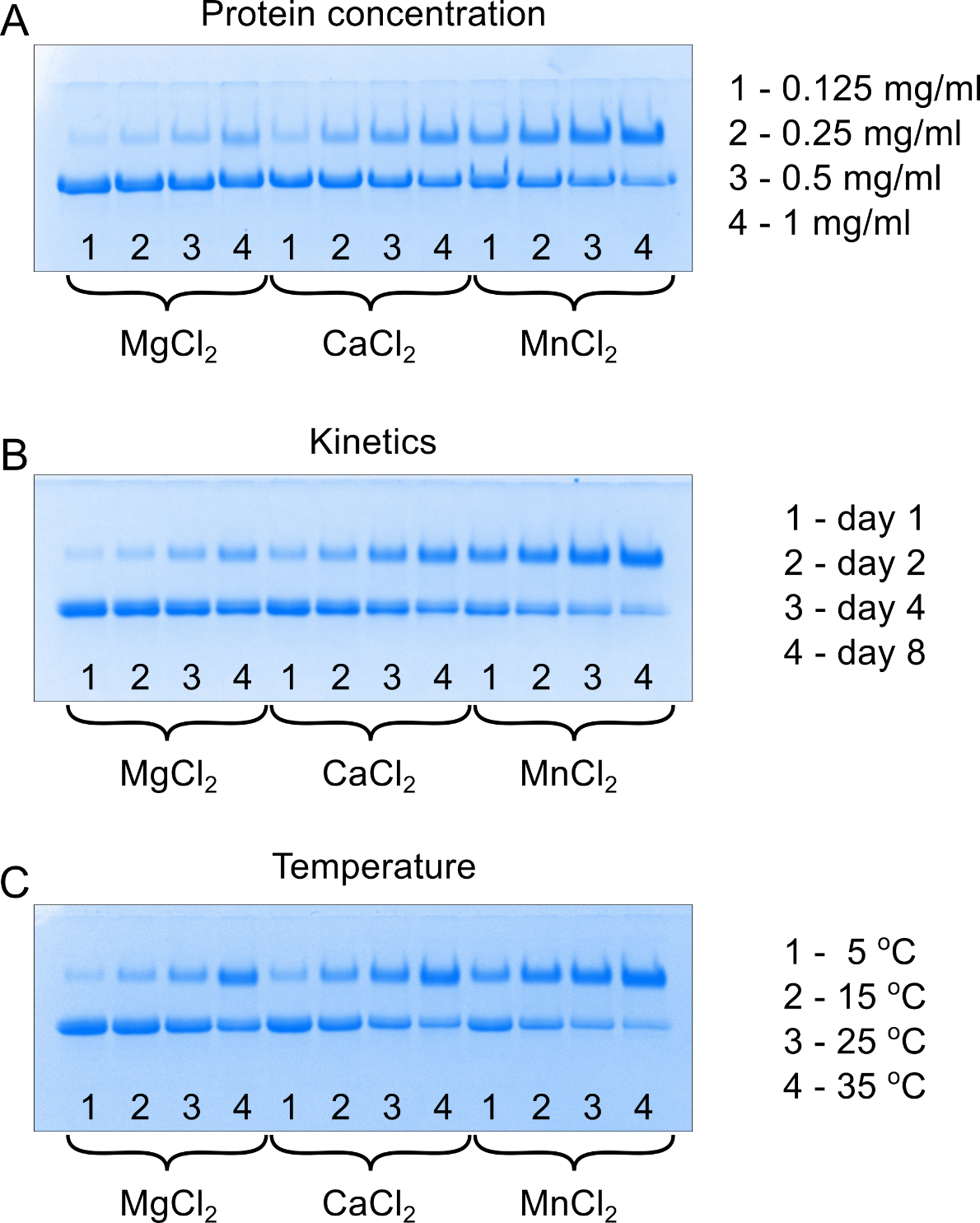


Figure S19. **Typical gels for analysis of the hexamer assembly under various conditions.** CVC trimer in 25 mM Tris-acetate buffer supplemented with 100 mM Na-acetate was used for hexamer assembly assays. (A) Variation of protein concentration. Protein at concentrations 0.125, 0.25. 0.5, and 1 mg/ml was subjected to assembly in the presence of 20 mM MgCl_2_, CaCl_2_, or MnCl_2_. at 25 °C for 5 days. (B) Kinetics of assembly. Protein at 1 mg/ml concentration was subjected to assembly in the presence of 20 mM MgCl_2_, CaCl_2_, or MnCl_2_ at 25 °C for 1, 2, 4, and 8 days. (C) Effect of temperature. Protein at 1 mg/ml concentration was subjected to assembly in the presence of 20 mM MgCl_2_, CaCl_2_, or MnCl_2_. at 5, 15, 25, and 35 °C for 7 days. The hexamer assembly was detected as the appearance of an upper SDS-resistant band. The samples were analyzed on 10% Bis-Tris SDS-PAGE with MOPS running buffer and coomassie stain.


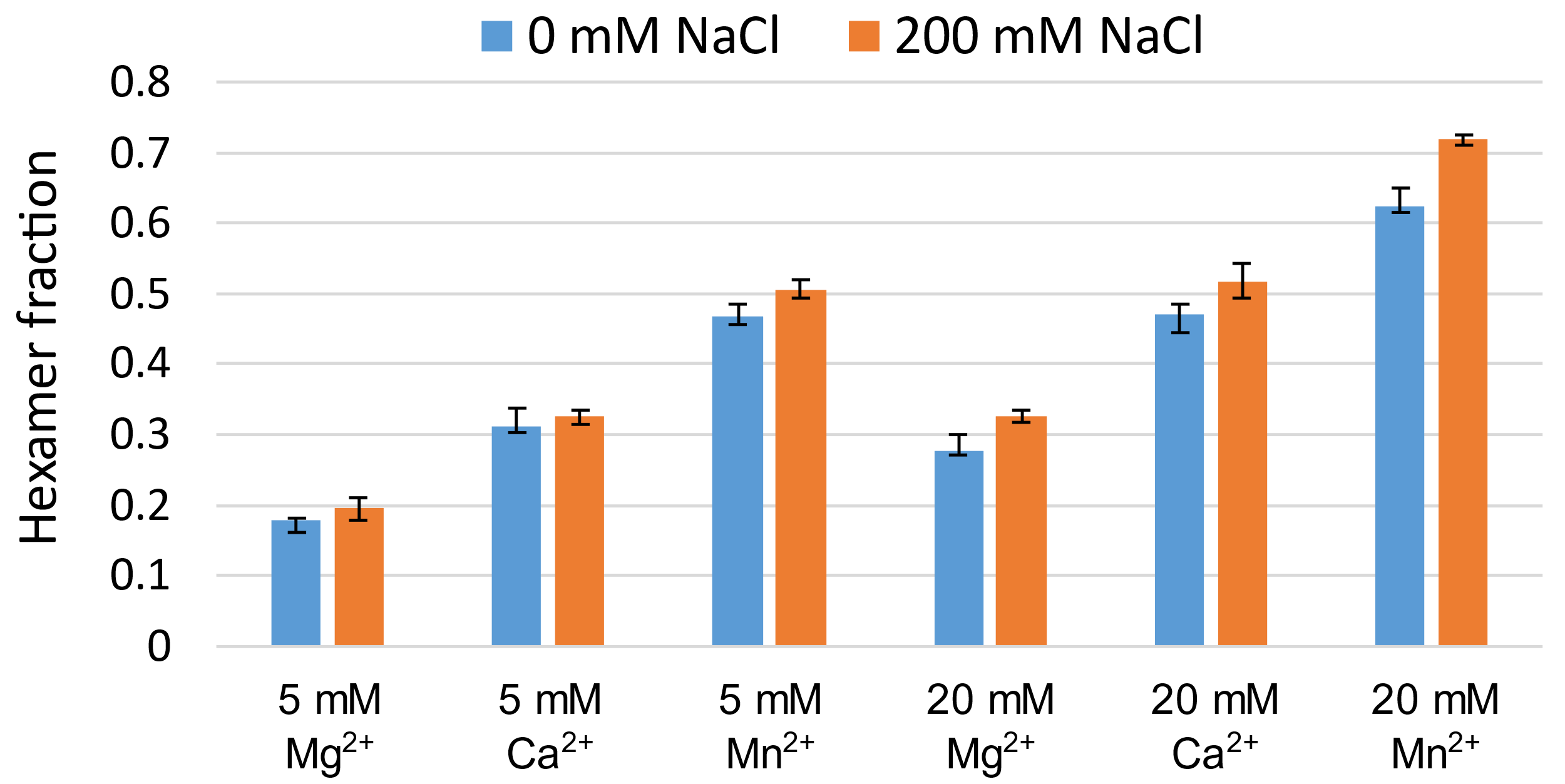


Figure S20. **Chloride effect on the hexamer assembly.** CVC trimer in Tris-acetate buffer supplemented with 100 mM Na-acetate was used for hexamer assembly assays in the absence or presence of 200 mM NaCl. Protein at 1 mg/ml concentration was subjected to assembly in the presence of 5 or 20 mM Mg-, Ca-, or Mn-acetate at 25 °C for 7 days.


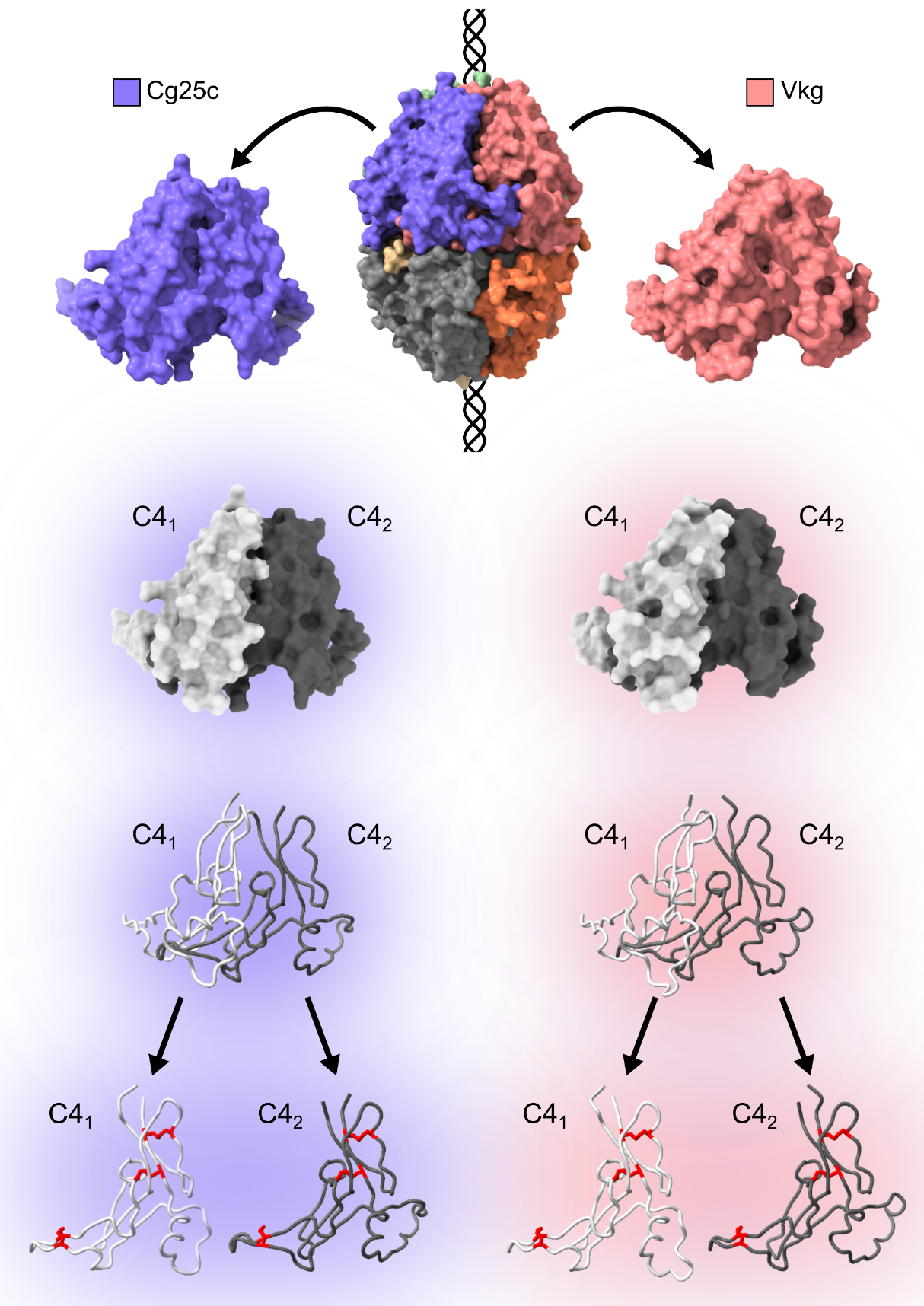


Figure S21. **Drosophila NC1 domain organization is similar to mammalian.** Individual Cg25c and Vkg chains are composed of two C4 subunits. Each C4 subunit is stabilized by three pairs of disulfide bonds shown in red.


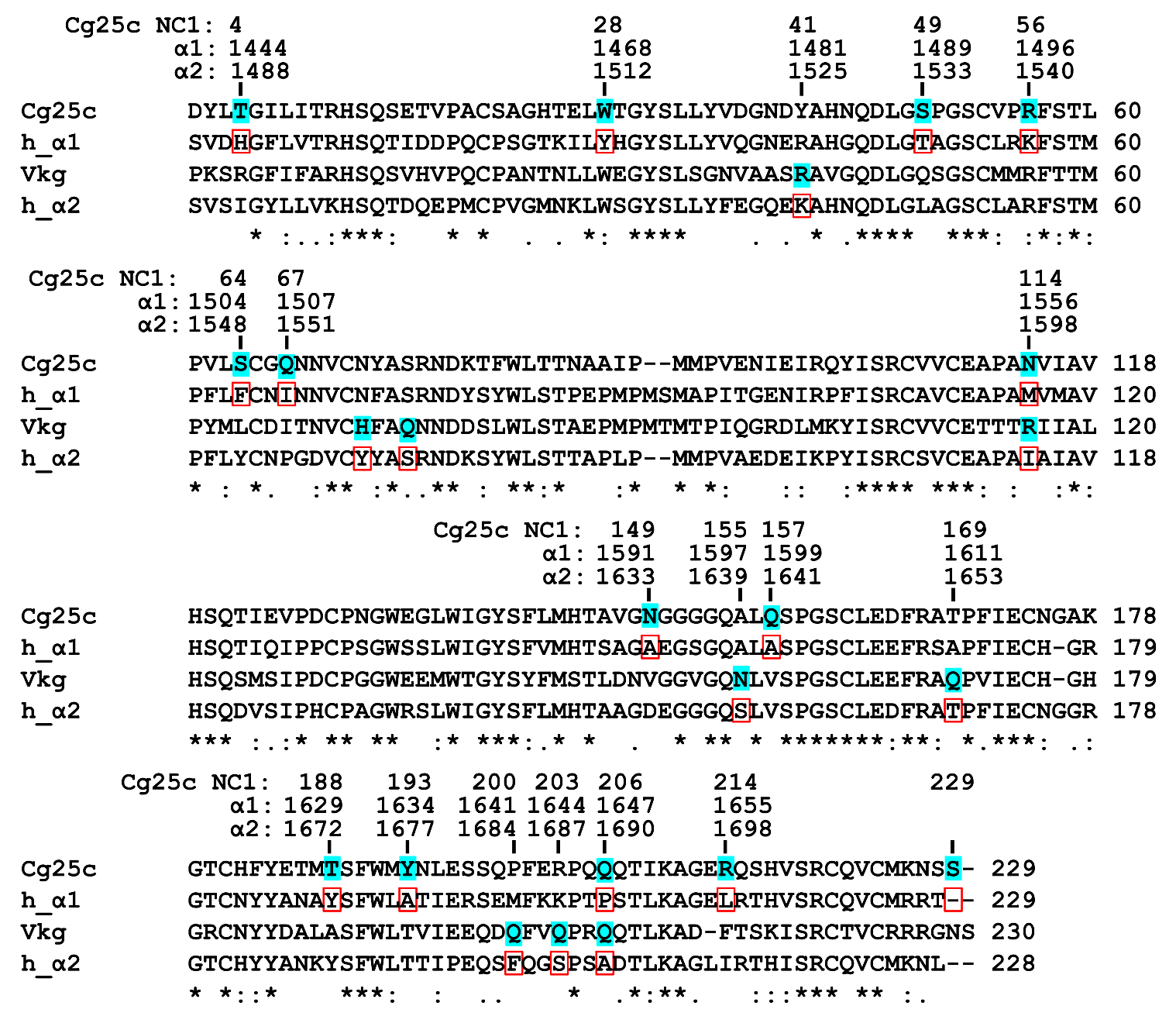


Figure S22. ***Drosophila*-unique residues involved in trimer association.** Sequence alignment of *Drosophila* and human NC1 chains. Cyan bars highlight those trimer-stabilizing residues in *Drosophila* sequences that are not found in the corresponding positions of human chains (red boxes). The numbering of residues is given for the NC1 domain and full-length sequences of the human α1 and α2 chains.


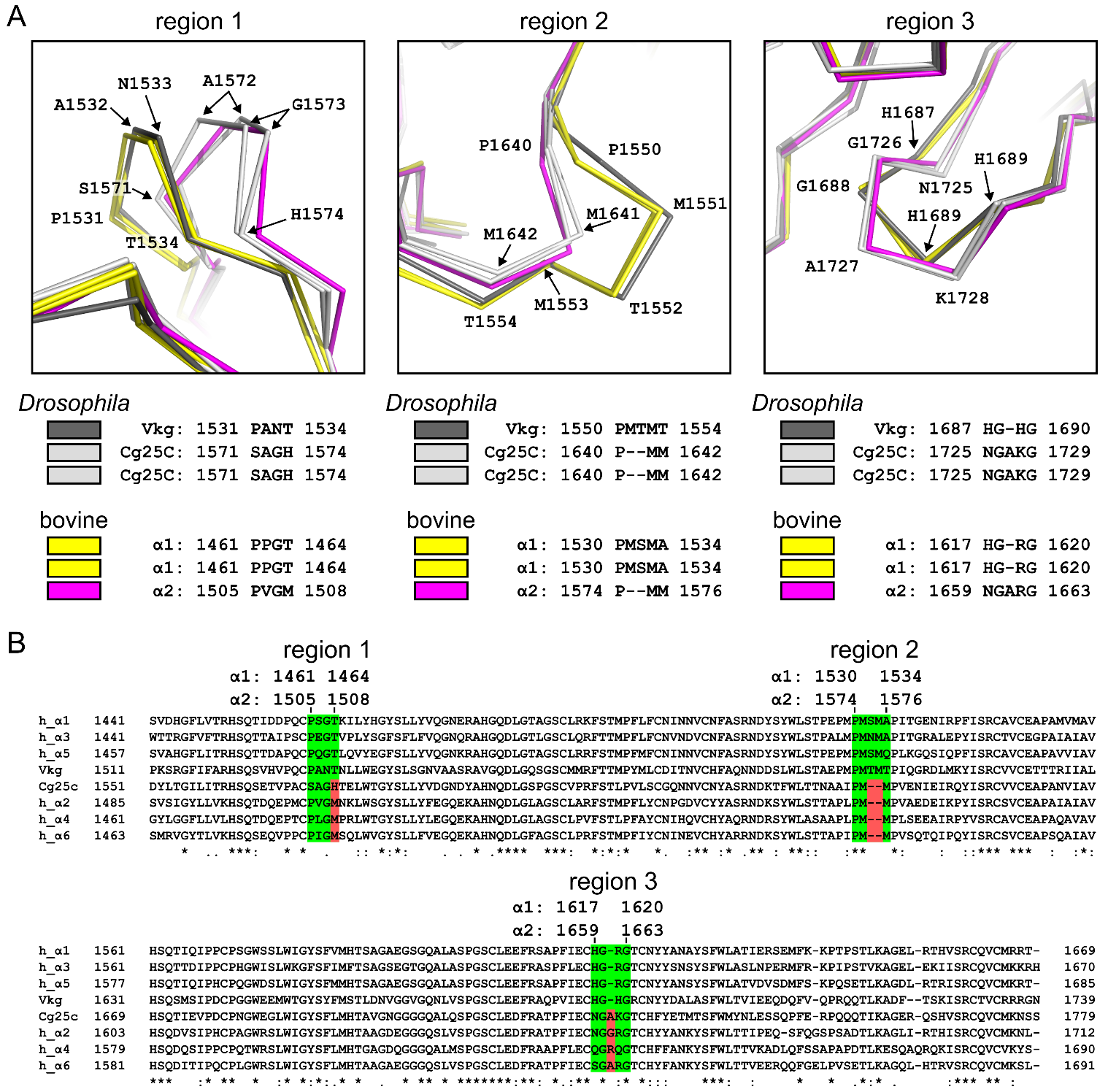


Figure S23. **Clustering of Drosophila and human NC1 chains.** (**A**) Three regions are structurally compared after the overall SSM superimposition of individual chains. In all three cases, the Vkg chain is structurally more homologous to the α1 chains, whereas the two Cg25c chains demonstrate a pattern of the α2 chain. For clarity only shown α1 and α2 chains of bovine NC1 domain (PDB ID 1t61). Originally, several bovine α1α1α2 and human α1α1α and α3α4α5 NC1 crystal structures were compared to reveal these structural patterns of α1, α3, and α5 chains as α1-like and α2 and α4 as α2-like chains. (**B**) Sequence alignment of NC1 chains. Green bars highlight these three regions that are structurally different between α1- and α2-like chains. Red bars point to key differences in α2-like chains, *i.e.*, in region 1 replacement of threonine to bulkier residues histidine and methionine, in region 2 deletion of two residues, and in region 3 insertion of one residue. Analysis of these regions in other animals will assist in chain clustering. The numbering of residues is given for the full-length *Drosophila* and human chains.


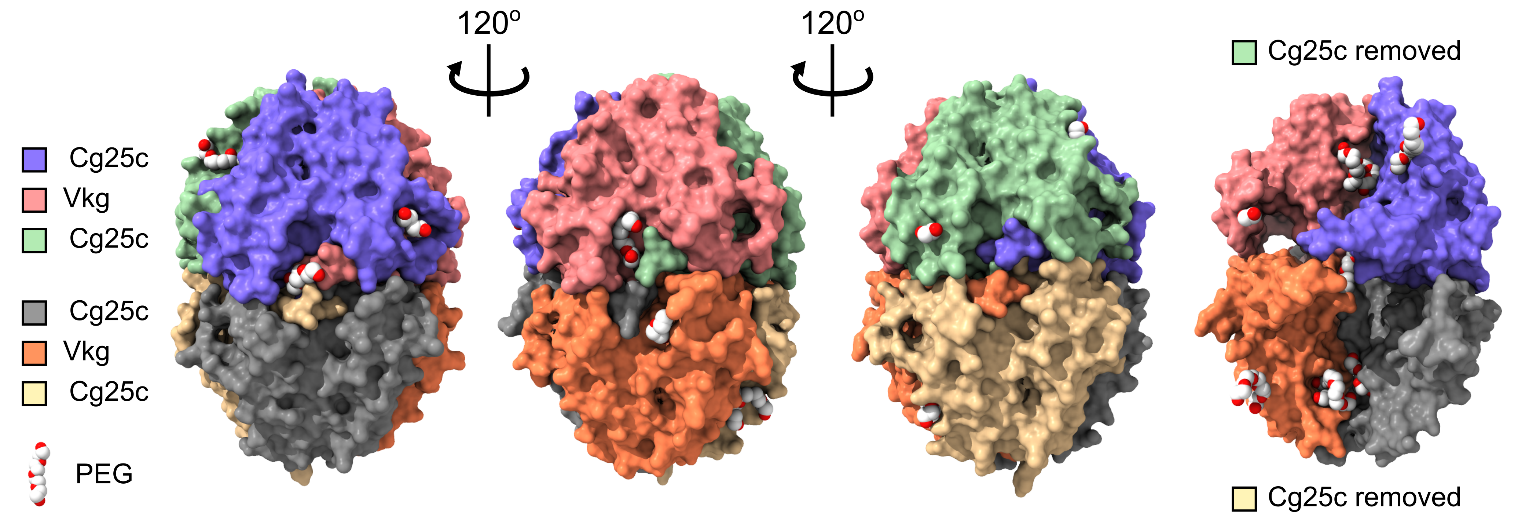


Figure S24. **Polyethylene glycol (PEG) molecules in the crystal structure.** Multiple PEGs were found on the surface and in a central cavity of the NC1 hexamer. Some PEGs are associated with more than one chain, thus revealing an opportunity for the design of molecules, which can exhibit a stabilizing effect for the trimer and/or the hexamer.


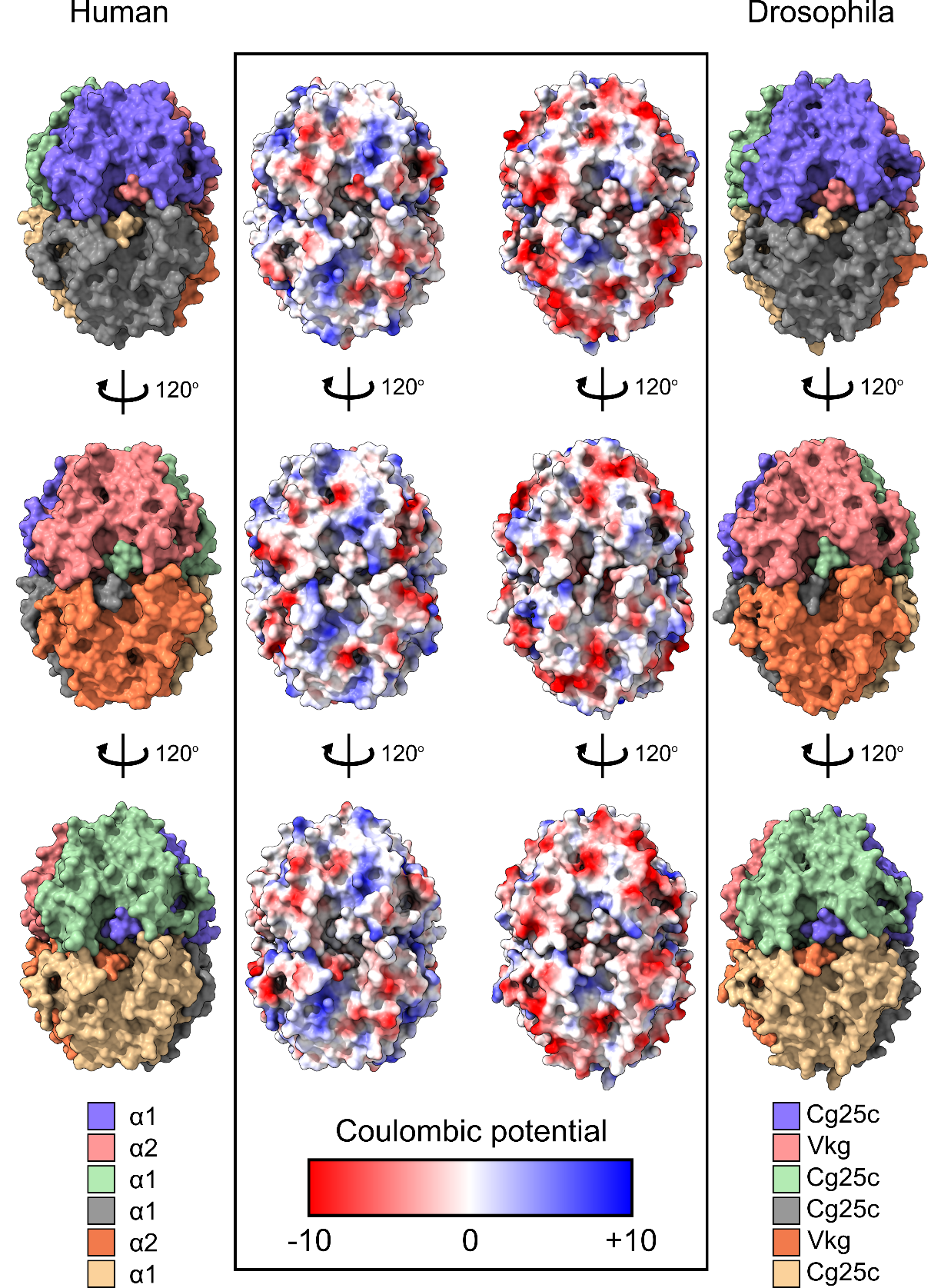


Figure S25. **Drosophila NC1 hexamer surface topology and electrostatic potential.** Coulombic electrostatic potentials were calculated with the ChimeraX program for *Drosophila* and human NC1 hexamers for comparison.


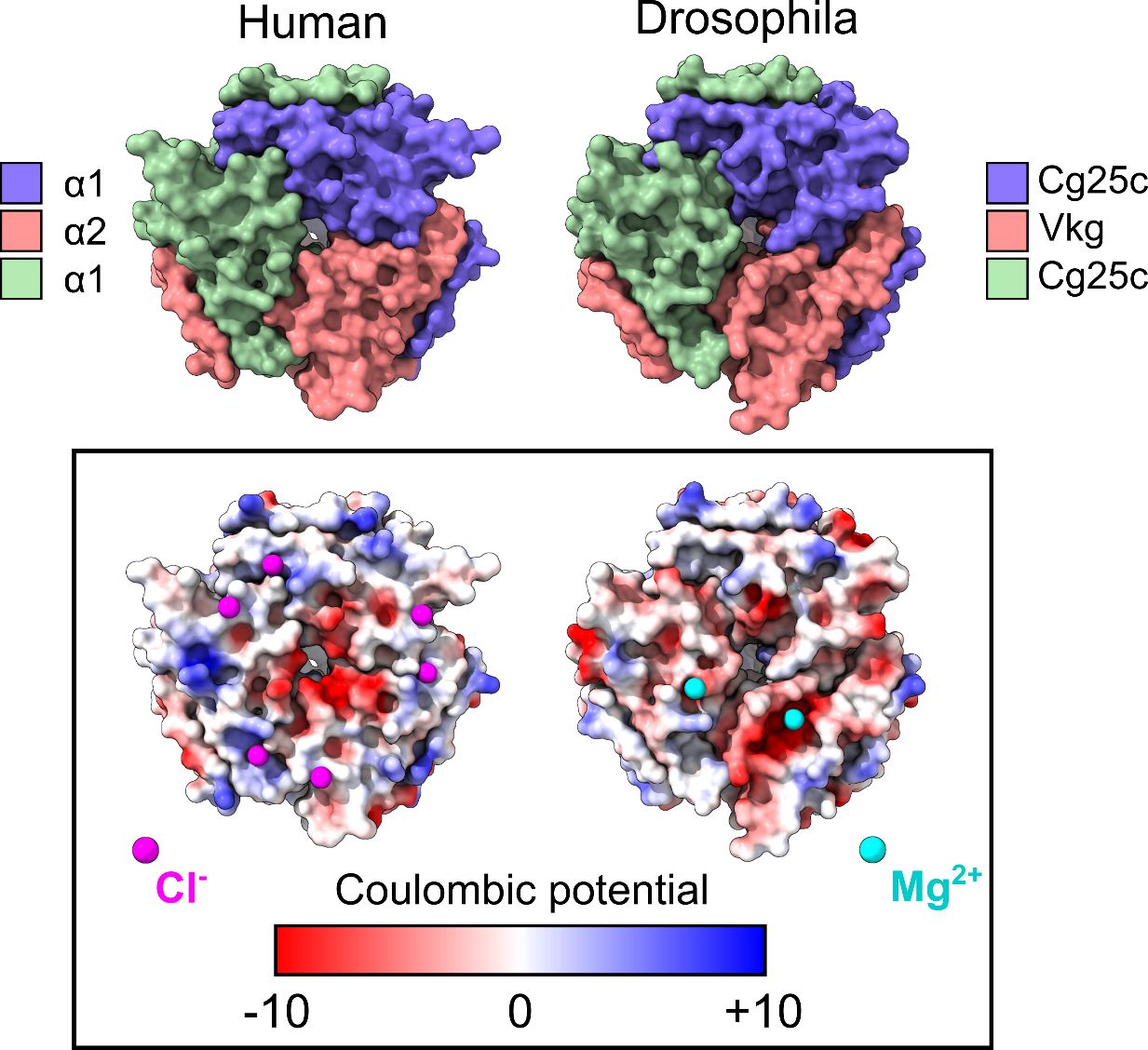


Figure S26. **Drosophila NC1 trimer surface topology and electrostatic potential.** Coulombic electrostatic potentials were calculated with the ChimeraX program for Drosophila and human NC1 hexamers for comparison.
